# The Zebrafish Cerebellar Neural Circuits Are Involved in Orienting Behavior

**DOI:** 10.1523/ENEURO.0141-24.2024

**Published:** 2024-10-24

**Authors:** Shiori Hosaka, Miu Hosokawa, Masahiko Hibi, Takashi Shimizu

**Affiliations:** Department of Biological Science, Graduate School of Science, Nagoya University, Nagoya, Aichi 464-8602, Japan

**Keywords:** autism spectrum disorders, cerebellum, orienting behavior, social behavior, zebrafish

## Abstract

Deficits in social behavior are found in neurodevelopmental disorders, including autism spectrum disorders (ASDs). Since abnormalities in cerebellar morphology and function are observed in ASD patients, the cerebellum is thought to play a role in social behavior. However, it remains unknown whether the cerebellum is involved in social behavior in other animals and how cerebellar circuits control social behavior. To address this issue, we employed zebrafish stereotyped orienting behavior as a model of social behaviors, in which a pair of adult zebrafish in two separate tanks approach each other, with one swimming at synchronized angles (orienting angles) with the other. We harnessed transgenic zebrafish that express botulinum toxin, which inhibits the release of neurotransmitters, in either granule cells or Purkinje cells (PCs), and zebrafish mutants of *reelin*, which is involved in the positioning of cerebellar neurons, including PCs. These zebrafish, deficient in the function or formation of cerebellar neural circuits, showed a significantly shorter period of orienting behavior compared with their control siblings. We found an increase in c-*fos* and *egr1* expression in the cerebellum after the orienting behavior. These results suggest that zebrafish cerebellar circuits play an important role in social orienting behavior.

## Significance Statement

Abnormalities in cerebellar morphology and function are often observed in autism spectrum disorder (ASD) patients. We describe the roles of cerebellar neural circuitry in social behavior using stereotyped orienting behavior in zebrafish, in which a pair of zebrafish in two separate tanks approach each other and show synchronous swimming. Neurotoxin-mediated inhibition of cerebellar neurons or mutations of the *reelin* gene, which is required for proper formation of cerebellar neural circuits, shortened the period of the orienting behavior. Furthermore, we found activation of the cerebellum in response to the orienting behavior. Our findings suggest that studies of zebrafish cerebellar neural circuits may provide a model for studying abnormalities in social behaviors, such as those seen in ASD.

## Introduction

Social behavior involves interactions between individual animals, important for their survival. Neurodevelopmental disorders, such as autism spectrum disorder (ASD), often exhibit deficits in social behavior. Although the exact causes of ASD are not fully understood, it is suggested that ASD results from a complex interplay of genetic and environmental factors. Brain regions involved in social behavior and communication, including the cerebellum, are affected in individuals with ASD (Bauman and Kemper, 2005; Mapelli et al., 2022).

The cerebellum is known for its role in motor coordination and motor learning, and it is also implicated in emotions and cognitive functions (Ito, 2005, 2006; Van Overwalle et al., 2014; Schmahmann, 2019). Recent studies highlight its role in social cognition, including recognizing and responding to social cues (Koziol et al., 2014; Van Overwalle et al., 2020). Its functions rely on conserved neural circuit structures in vertebrates (Hashimoto and Hibi, 2012; Hibi and Shimizu, 2012; Hibi et al., 2017). Purkinje cells (PCs) and granule cells (GCs) are the major GABAergic and glutamatergic neurons in the cerebellum, respectively, each receiving two types of inputs from outside the cerebellum. PCs receive climbing fibers from the inferior olivary nuclei, while GCs receive mossy fibers (MFs) from various brain regions. The information from MFs is conveyed by GC axons. PCs integrate these two inputs and send outputs through projection neurons, which are deep cerebellar nucleus neurons in mammals and eurydendroid cells in teleosts.

Accumulating evidence suggests a link between cerebellar abnormalities and ASD. For example, ASD symptoms are associated with reduced cerebellar structure (Pierce and Courchesne, 2001; Whitney et al., 2008; Riva et al., 2013; D'Mello et al., 2015). Cerebellar injury or lesions in preterm infants (van der Heijden et al., 2021) or children (Levisohn et al., 2000; Korah et al., 2010; Boswinkel et al., 2019; Gill and Sillitoe, 2019) and cerebellar malformations are linked to ASD-like symptoms (Bolduc et al., 2012). Mouse models of autism with mutations in ASD-related genes (*Cadps2*, *Gabrb3*, *Shank3*, *Mecp2*, and *Cntnap2*) often exhibit cerebellar abnormalities like hypoplasia (Sadakata et al., 2007; DeLorey et al., 2008; Kloth et al., 2015; Mapelli et al., 2022). A PC-specific deficiency of the tuberous sclerosis complex 1 (*TSC1*) gene, associated with a genetic disorder with high ASD comorbidity, in mice resulted in ASD-like behaviors (Tsai et al., 2012). In this model, the neural circuit from the cerebellum to the medial PFC was implicated in the ASD-like symptoms (Kelly et al., 2020). The projection from the cerebellum to the ventral tegmental area (VTA) is required for mice to exhibit social behavior (Carta et al., 2019). These reports strongly suggest a link between cerebellar dysfunction and social deficits. However, the mechanisms by which cerebellar neural circuits control social behaviors and the extent to which cerebellar dysfunction contributes to social deficits in other vertebrate species remain unknown.

Zebrafish, an ideal model for studying cerebellar neural circuits in social behavior, offer various transgenic (Tg) lines expressing genes in these circuits and mutants with cerebellar defects (Bae et al., 2009; Takeuchi et al., 2015a, b; Nimura et al., 2019; Itoh et al., 2020, 2021). Zebrafish are highly social animals that exhibit diverse social behaviors (Suriyampola et al., 2016; Orger and de Polavieja, 2017). Adult and larval zebrafish pairs exhibit orienting behavior, in which they are spatially separated and can view each other through a divider; they approach and behave in a stereotyped orienting pattern (Dreosti et al., 2015; Stednitz et al., 2018; Stednitz and Washbourne, 2020). A similar type of zebrafish social behavior was used to examine an ASD-risk gene (Ruzzo et al., 2019). Zebrafish also show a preference for interacting with unfamiliar fish (Dreosti et al., 2015), and mutants of genes related to Reelin (Reln) signaling, associated with certain ASD (Lammert et al., 2017; Sanchez-Sanchez et al., 2018; Hernandez-Garcia et al., 2020; Nawa et al., 2020; Scala et al., 2022), showed defects in this social novelty preference (Dalla Vecchia et al., 2019). These findings make zebrafish social behaviors valuable for understanding the neural circuits and genes involved in social behaviors and ASD etiology. The *lhx8a*-expressing cholinergic neurons in the ventral forebrain and the immediate early gene *egr1*, required for forebrain dopaminergic signaling, are shown to be involved in zebrafish orienting behaviors (Stednitz et al., 2018; Tallafuss et al., 2022). It is still unclear whether the cerebellum is also involved in this behavior.

In this study, we investigated the roles and activities of the cerebellum in orienting behavior by using zebrafish.

## Materials and Methods

### Ethics statement

The animal experiments in this study were approved by the Institutional Animal Experiment Committee and were conducted in accordance with the Regulations on Animal Experiments at the institute.

### Zebrafish and medaka

**Figure 1.**
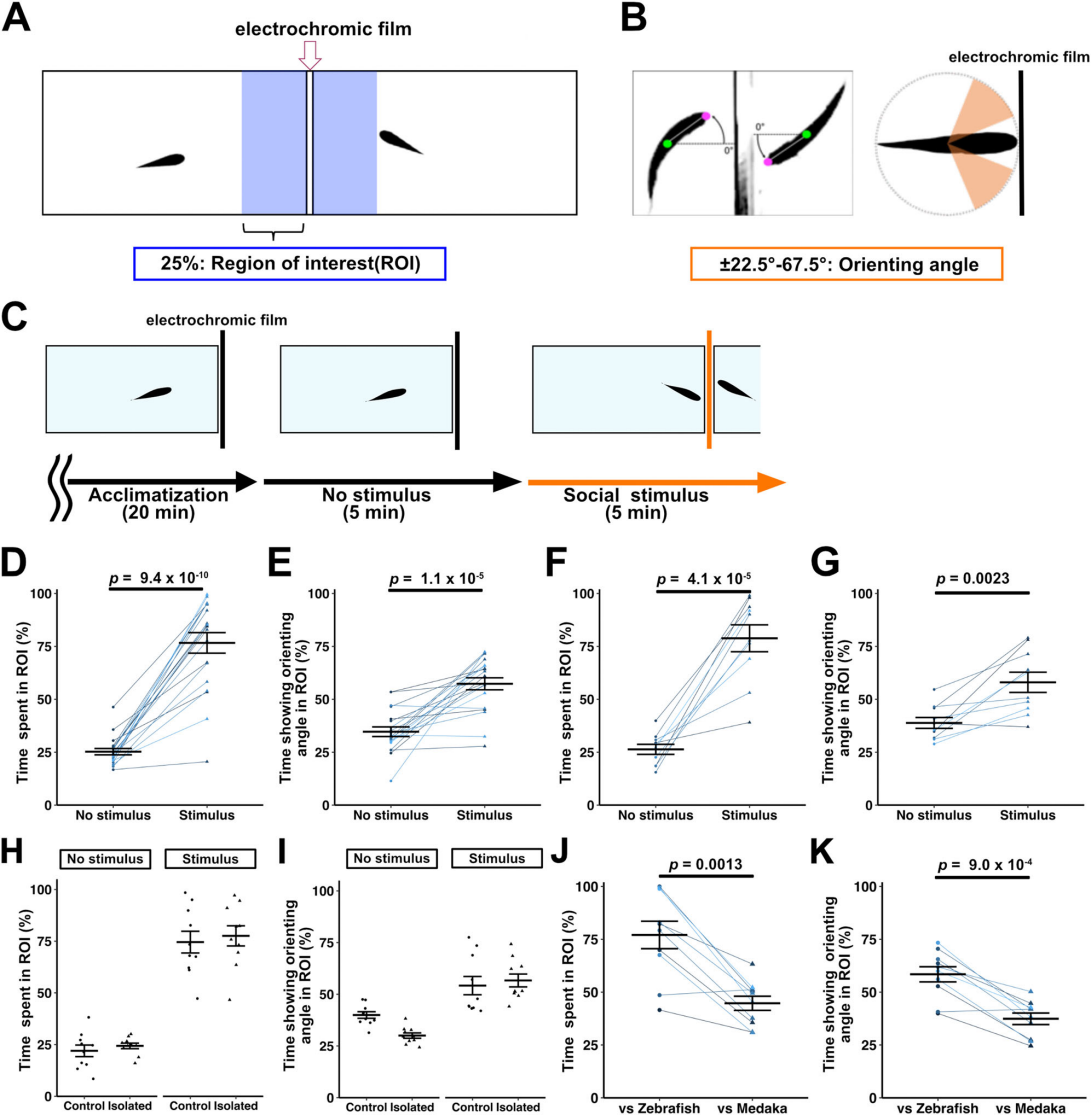
Orienting behavior of zebrafish toward conspecifics. ***A***, Apparatus for orienting behavior. Two large tanks (L 9 × W 18 × H 6 cm, filled to 5.7 cm with water) were placed across a divider made of a light-dimming electrochromic film. The 25% area of the tank divider side was set as the ROI. ***B***, Orienting angle. Behavior of looking at fish in the opposite tank at the head angle ±22.5–67.5° (orienting angle) from the direction perpendicular to the divider was considered orienting behavior. ***C***, Time course of orienting behavior assay. Zebrafish were first placed in the tanks and allowed to swim *ad libitum* for 20 min to acclimate to the tanks (acclimatization). The swimming behavior was then recorded for 5 min when the divider was opaque (no-stimulus), followed by 5 min of swimming behavior when the divider was transparent (social stimulus). ***D***, The percentage of the time in which zebrafish stayed in the ROI under no-stimulus or social stimulus conditions. ***E***, The percentage of the time in which zebrafish exhibited the orienting angle in the ROI under no-stimulus or social stimulus conditions. Wild-type zebrafish stayed in the ROI and showed the orienting angles for a significantly longer period under the stimulus condition compared with the no-stimulus condition (*n *= 20 each in ***D***; *n *= 20 each in ***E***). ***F***, ***G***, The percentage of the time spent in the ROI and the percentage of the time that fish show the orienting angle when using a combination of standard (L 9 × W 18 × H 6 cm) and short (L 9 × W 9 × H 6 cm) tanks. Zebrafish behavior in the large tanks was analyzed. They stayed in the ROI and showed the orienting angle for a longer period under the stimulus condition than the no-stimulus condition (*n *= 10 each in ***F***; *n *= 10 each in ***G***). ***H***, ***I***, The percentage of the time spent in ROI and the percentage of the time showing orienting angles for isolated zebrafish reared with no other fish in sight until adulthood. Two large tanks were used. Isolated zebrafish stayed in the ROI and showed the orienting angles comparable with those of control zebrafish (*n *= 10 each in ***H***; *n *= 10 each in ***I***). ***J***, ***K***, Zebrafish orienting behaviors toward medaka. After analyzing the orienting behavior of zebrafish toward zebrafish, the orienting behavior of zebrafish toward medaka was analyzed. When medaka was used as a stimulus, zebrafish stayed in the ROI and showed the orienting angle for less time than when zebrafish was used as stimulus (*n *= 10 in ***J***; *n *= 10 in ***K***). See Extended Data Figure 1-1 for more details.

Wild-type zebrafish with the Oregon AB genetic background and four previously reported Tg lines, *gSA2AzGFF152B* (Takeuchi et al., 2015b), which expresses a modified version of Gal4-VP16 (GAL4FF, also referred to as GFF) in the corpus cerebelli GCs; *Tg(cbln12:Gal4FF)*, which expresses GFF in the cerebellar GCs (Koyama et al., 2021); *Tg(UAS:BoTxBLC-GFP)^icm21^*, which expresses the light chain of botulinum toxin (BoTx) in a GAL4-dependent manner (Sternberg et al., 2016); and *Tg(aldoca:BoTxBLC-GFP)*, which expresses the light chain of BoTx in the cerebellar PCs (Koyama et al., 2021), were used. Fish were obtained from crosses between Tg lines or from crosses with the AB line. At 5 d post fertilization, expression of GFP was examined under a fluorescence dissection microscope, and fish with confirmed GFP expression were used for analyses. Sibling fish that did not express GFP were designated as control fish. For the zebrafish mutant, the *reln^nub23^* allele, which contains a 7 bp deletion in the second Reln repeat and is likely a null allele (Nimura et al., 2019), was used. To obtain homozygous mutant fish, heterozygous mutant male and female fish were crossed. Homozygotes and heterozygotes (as control) were identified by genotyping. For orienting behavior analyses, 4–6-month-old adults were used. Fish were maintained in a 14–10 h light/dark cycle (light from 9 A.M. to 11 P.M.; dark from 11 P.M. to 9 A.M.) at 28.5°C. Some experiments (Fig. 1) used quarantined zebrafish, which were raised with no other fish in sight until they reached adulthood. To raise quarantined zebrafish, we placed fertilized eggs one by one in paper cups and have grown zebrafish for 1 month in paper cups and subsequently in large tanks wrapped in aluminum foil. The other zebrafish were raised in a rearing tank (3 L) with 10–20 siblings until they reached adulthood. In some experiments Fig. 1), the Nagoya strain of the medaka fish *Oryzias latipes* was used as stimuli of the social behavior assay. We used eight male and two female medaka fish. The sizes of medaka and zebrafish were 3.01 ± 0.03 and 4.01 ± 0.05 cm, respectively. All experiments were conducted without distinction between males and females.

### Genotyping

Tail fins of adult fish were cut and incubated in DNA extraction buffer (10 mM Tris–HCl, 2 mM EDTA, 0.2% Triton X-100), pH 8.0, with 0.4 mg/ml proteinase K at 55°C for 2 h. After incubation, proteinase *K* was inactivated at 99°C for 10 min. The resulting DNA solution was used as a template for PCR. Primers for detection of the BoTx and *Gal4FF* gene and mutation in *reln^Δ7^* were listed in Table 1. The PCR products were separated on 12% TBE (Tris-borate-EDTA) acrylamide gels (for deletion mutants) or 1% TAE (Tris-acetate-EDTA) agarose gels (for transgenes).

**Table 1.** Summary of PCR primers

Gene	Primer
Gal4FF	5′-AAGTGCGCCAAGTGTCTGAAGAAC-3′
	5′-GACCTGGACATGCTGCCTGCTGAT-3′
BoTx	5′-CGAACATAGCTAGCGTGACCGTGA-3′
	5′- TGGAGCACGTGTATCAGCTCATGC –3′
*reelin^Δ7^*	5′- CGTTTCCGCTGGATCCAGA-3′
	5′-CGTGGCACATCTGTGGACA-3′
c-*fos*	5′- ACCCGAGCTCTTACCCCAAAATG-3′
	5′-CTTGCAGATGGGTTTGTGTGCG-3′
*egr-1*	5′-TTCTCTCCTCTCCGAGCACACT-3′
	5′- AGCCGTTGGGTGGAGTAGGTCT-3′
*gapdh*	5′-TGTGGTTGTGTCTGCTCCATC-3′
	5′-ATCGACAGTCTTCTGTGTGG-3′

### Social behavior assay

Social behavior assays were conducted based on the previous report (Stednitz et al., 2018). Briefly, adult zebrafish were moved from the fish facility to the laboratory the day before the assay, transferred to individual tanks, and a white paper was placed between each tank so that the fish could not see each other. Behavior assays were conducted in two transparent tanks (standard tanks, L 9 × W 18 × D 6 cm, filled to 5.7 cm with water) placed across a divider. In some experiments, a short tank (L 9 × W 9 × D 6 cm) was used in combination with the large tank, with stimulus fish in the short tank. All sides except the divider side of the tanks were transparent. The divider was made of a light-dimming electrochromic film (Surprised Glass, Able) attached to a 2-mm-thick acrylic plate. The electrochromic film was then switched on to become transparent. The behavior apparatus was illuminated from above using white LED lights. Tanks were placed on a clear acrylic plate and imaged from below with a video camera (USB 3.0 camera FLIR FL3-U3-13E4 B/W, Edmund Optics) at 30 fps. Individual fish were placed one by one in the tanks with clean fish water at a depth of 5.7 cm and allowed to swim *ad libitum* for 20 min to acclimate to the tanks. The swimming behavior was then recorded for 5 min when the divider was opaque and not visible to each other (no-stimulus condition), followed by 5 min of swimming behavior when the divider was transparent and visible to each other (stimulus condition). The 25% of the tank divider side was set as the region of interest (ROI; Fig. 1*A*). Behavior looking at fish in the opposite tank at the head angle ±22.5–67.5° (orienting angle) from the direction perpendicular to the divider was considered orienting behavior (Fig. 1*B*). The timing of making the divider transparent was controlled by a USB DAQ device (USB-6001; National Instruments) and a program created in LabVIEW (National Instruments). The position of the head, center, and tail and the head angle of each individual were analyzed from videos of zebrafish swimming behavior using the tracking software Fish Tracker (Abril-de-Abreu et al., 2015). The number of frames in which the *x*-coordinate of the center of the fish was in the ROI and the number of frames in which fish exhibited an orienting angle were counted using R (version 3.6.1). The percentage of the time the fish swam in the ROI and the percentage of the time the fish exhibited an orienting angle during the 5 min swimming were calculated. The distance between the centers of fish between two consecutive frames was calculated using R. An event showing >90° in the head angle change of two consecutive movements was counted as a turning. The average swimming speed and turning frequency were determined.

### RT-qPCR

After the social behavior assays (swimming in 5 min no-stimulus and 5 min stimulus conditions), the fish were left under no-stimulus conditions for 15 min to allow time for transcription of c-*fos* (*fosab* in ZFIN, https://zfin.org) and *egr1* mRNA. The fish were transferred from the tank into ice water and placed for at least 3 min before decapitation. RNA extraction and RT-qPCR were performed as reported previously (Itoh et al., 2021). The cerebellum was removed in PBS (137 mM NaCl, 8.1 mM Na_2_HPO_4_, 2.7 mM KCl, 1.5 mM KH_2_PO_4_), pH 7.4, and each individual sample was placed in a separate tube. After adding 100 μl of TRIzol LS reagent (Thermo Fisher Scientific, catalog #15596026), the cerebellum was thoroughly crushed in a homogenizer ULTRA TURRAX T25 basic (IKA). To extract RNA, 100 μl of chloroform was added, and the aqueous phase was harvested after centrifugation. RNA was precipitated by adding 250 μl of isopropanol and further centrifugation. A 150 ng of RNA from the cerebellum of each individual was used for cDNA synthesis using ReverTra Ace and oligo-dT 20 primers (Toyobo) according to the manufacturer's protocol. Using the synthesized cDNA as a template, qPCR was performed with THUNDERBIRD Next SYBR qPCR Mix (Toyobo) and LightCycler Nano (Roche Diagnostics) to amplify c-*fos, egr-1* and the housekeeping gene glyceraldehyde-3-phosphate dehydrogenase (*gapdh*; Wu et al., 2019). The primers used for qPCR were listed in Table 1. The ggplot2 package in R was used to create the graphs.

### Statistics

Data were analyzed using Microsoft Excel (version 16.71) and R (version 4.2.2). Results are presented as mean ± standard error of the mean (SEM). Statistical procedures used were the two-way mixed ANOVA with Tukey's post hoc test, paired *t* test, Brunner–Munzel test, and Welch's *t* test. Effect sizes were measured using *η*^2^, Cliff's Delta, and Cohen's *d* with Hedges’ correction. The statistical details are presented in Table 2.

**Table 2.** Summary of statistical analyses

Figures	Measurement	Type of test	Comparison	Statistical value	Effect size
1*D*	Time spent in ROI (%)	Paired *t* test	No-stimulus versus stimulus	*p *= 9.4 × 10^−10^; *t*_(19)_ = 11	Cohen's *d* (Hedges’ *g*) = 3.1
1*E*	Time showing orienting angle in ROI (%)	Paired *t* test	No-stimulus versus stimulus	*p *= 1.1 × 10^−5^; *t*_(19)_ = 5.9	Cohen's *d* (Hedges’ *g*) = 1.9
1*F*	Time spent in ROI (%)	Paired *t* test	No-stimulus versus stimulus	*p *= 4.1 × 10^−5^; *t*_(9)_ = 7.4	Cohen's *d* (Hedges’ *g*) = 3.3
1*G*	Time showing orienting angle in ROI (%)	Paired *t* test	No-stimulus versus stimulus	*p *= 0.0023; *t*_(9)_ = 4.2	Cohen's *d* (Hedges’ *g*) = 1.5
1*H*	Time spent in ROI (%)	Two-way mixed ANOVA	Social experiences × social stimulus interaction	*p *= 0.94; *F*_(1,18) _= 0.007	*η*^2 ^= 3.1 × 10^−5^
1*H*	Time spent in ROI (%)	Two-way mixed ANOVA	Main effect of social experiences	*p *= 0.49; *F*_(1,18) _= 0.49	*η*^2 ^= 2.2 × 10^−3^
1*H*	Time spent in ROI (%)	Two-way mixed ANOVA	Main effect of social stimulus	*p *= 7.8 × 10^−11^; *F*_(1,18)_ = 181	*η*^2 ^= 0.83
1*I*	Time showing orienting angle in ROI (%)	Two-way mixed ANOVA	Social experiences × social stimulus interaction	*p *= 0.041; *F*_(1,18) _= 4.9	*η*^2 ^= 0.050
1*I*	Time showing orienting angle in ROI (%)	Two-way mixed ANOVA	Main effect of social experiences	*p *= 0.23; *F*_(1,18) _= 1.6	*η*^2 ^= 0.018
1*I*	Time showing orienting angle in ROI (%)	Two-way mixed ANOVA	Main effect of social stimulus	*p *= 9.4 × 10^−7^; *F*_(1,18) _= 53	*η*^2 ^= 0.54
1*I*	Time showing orienting angle in ROI (%)	Tukey's post hoc test	Control fish with no-stimulus versus control fish with stimulus	*p *= 0.0070	Cohen's *d* (Hedges’ *g*) = 1.3
1*I*	Time showing orienting angle in ROI (%)	Tukey's post hoc test	Isolated fish with no-stimulus versus isolated fish with stimulus	*p *= 8.0 × 10^−7^	Cohen's *d* (Hedges’ *g*) = 3.4
1*I*	Time showing orienting angle in ROI (%)	Tukey's post hoc test	Control fish with no-stimulus versus isolated fish with no-stimulus	*p *= 0.089	Cohen's *d* (Hedges’ *g*) = −2.1
1*I*	Time showing orienting angle in ROI (%)	Tukey's post hoc test	Control fish with stimulus versus isolated fish with stimulus	*p *= 0.93	Cohen's *d* (Hedges’ *g*) = 0.20
1*J*	Time spent in ROI (%)	Paired *t* test	Zebrafish versus medaka	*p *= 0.0013; *t*_(9)_ = 4.6	Cohen's *d* (Hedges’ *g*) = 1.9
1*K*	Time showing orienting angle in ROI (%)	Paired *t* test	Zebrafish versus medaka	*p *= 9.0 × 10^−4^; *t*_(9)_ = 4.9	Cohen's *d* (Hedges’ *g*) = 2.0
1-1*A*	Time spent in ROI (%)	Two-way mixed ANOVA	Sex × social stimulus interaction	*p *= 0.26; *F*_(3,26) _= 1.4	*η*^2 ^= 0.024
1-1*A*	Time spent in ROI (%)	Two-way mixed ANOVA	Main effect of sex	*p *= 0.29; *F*_(3,26) _= 1.3	*η*^2 ^= 0.037
1-1*A*	Time spent in ROI (%)	Two-way mixed ANOVA	Main effect of social stimulus	*p *= 3.5 × 10^−10^; *F*_(1,26) _= 95	*η*^2 ^= 0.54
1-1*B*	Time showing orienting angle in ROI (%)	Two-way mixed ANOVA	Sex × social stimulus interaction	*p *= 0.14; *F*_(3,26) _= 2.0	*η*^2 ^= 0.044
1-1*B*	Time showing orienting angle in ROI (%)	Two-way mixed ANOVA	Main effect of sex	*p *= 0.13; *F*_(3,26) _= 2.0	η^2 ^= 0.076
1-1*B*	Time showing orienting angle in ROI (%)	Two-way mixed ANOVA	Main effect of social stimulus	*p *= 1.9 × 10^−7^; *F*_(1,26) _= 49	*η*^2 ^= 0.37
2*E*	Time spent in ROI (%)	Two-way mixed ANOVA	BoTx × social stimulus interaction	*p *= 0.018; *F*_(1,38) _= 6.2	*η*^2 ^= 0.029
2*E*	Time spent in ROI (%)	Two-way mixed ANOVA	Main effect of BoTx	*p *= 0.033; *F*_(1,38) _= 4.9	*η*^2 ^= 0.022
2*E*	Time spent in ROI (%)	Two-way mixed ANOVA	Main effect of social stimulus	*p *= 9.5 × 10^−14^; *F*_(1,38) _= 128	*η*^2 ^= 0.60
2*E*	Time spent in ROI (%)	Tukey's post hoc test	Control fish with no-stimulus versus control fish with stimulus	*p *< 10^−16^	Cohen's *d* (Hedges’ *g*) = 3.3
2*E*	Time spent in ROI (%)	Tukey's post hoc test	152B::BoTx with no-stimulus versus 152B::BoTx with stimulus	*p *= 1.0 × 10^−7^	Cohen's *d* (Hedges’ *g*) = 1.9
2*E*	Time spent in ROI (%)	Tukey's post hoc test	Control fish with no-stimulus versus 152B::BoTx with no-stimulus	*p *= 1.0	Cohen's *d* (Hedges’ *g*) = 0.10
2*E*	Time spent in ROI (%)	Tukey's post hoc test	Control fish with stimulus versus 152B::BoTx with stimulus	*p *= 0.0072	Cohen's *d* (Hedges’ *g*) = −0.84
2*F*	Time showing orienting angle in ROI (%)	Two-way mixed ANOVA	BoTx × social stimulus interaction	*p *= 0.10; *F*_(1,38) _= 2.8	*η*^2 ^= 0.024
2*F*	Time showing orienting angle in ROI (%)	Two-way mixed ANOVA	Main effect of BoTx	*p *= 0.0047; *F*_(1,38) _= 9.0	*η*^2 ^= 0.060
2*F*	Time showing orienting angle in ROI (%)	Two-way mixed ANOVA	Main effect of social stimulus	*p *= 2.9 × 10^−7^; *F*_(1,38) _= 39	*η*^2 ^= 0.33
2*G*	Time spent in ROI (%)	Two-way mixed ANOVA	BoTx × social stimulus interaction	*p *= 0.0084; *F*_(1,42) _= 7.7	*η*^2 ^= 0.037
2*G*	Time spent in ROI (%)	Two-way mixed ANOVA	Main effect of BoTx	*p *= 0.042; *F*_(1,42) _= 4.4	*η*^2 ^= 0.018
2*G*	Time spent in ROI (%)	Two-way mixed ANOVA	Main effect of social stimulus	*p *= 8.5 × 10^−14^ *F*_(1,42) _= 118	*η*^2 ^= 0.57
2*G*	Time spent in ROI (%)	Tukey's post hoc test	Control fish with no-stimulus versus control fish with stimulus	*p *< 10^−16^	Cohen's *d* (Hedges’ *g*) = 4.3
2*G*	Time spent in ROI (%)	Tukey's post hoc test	cbln12::BoTx with no-stimulus versus cbln12::BoTx with stimulus	*p *< 10^−16^	Cohen's *d* (Hedges’ *g*) = 1.5
2*G*	Time spent in ROI (%)	Tukey's post hoc test	Control fish with no-stimulus versus cbln12::BoTx with no-stimulus	*p *= 0.93	Cohen's *d* (Hedges’ *g*) = 0.23
2*G*	Time spent in ROI (%)	Tukey's post hoc test	Control fish with stimulus versus cbln12::BoTx with stimulus	*p *= 0.0047	Cohen's *d* (Hedges’ *g*) = −0.88
2*H*	Time showing orienting angle in ROI (%)	Two-way mixed ANOVA	BoTx × social stimulus interaction	*p *= 9.8 × 10^−4^; *F*_(1,42) _= 13	*η*^2 ^= 0.056
2*H*	Time showing orienting angle in ROI (%)	Two-way mixed ANOVA	Main effect of BoTx	*p *= 0.054; *F*_(1,42) _= 3.9	*η*^2 ^= 0.026
2*H*	Time showing orienting angle in ROI (%)	Two-way mixed ANOVA	Main effect of social stimulus	*p *= 1.02 × 10^−12^; *F*_(1,42) _= 100	*η*^2 ^= 0.45
2*H*	Time showing orienting angle in ROI (%)	Tukey's post hoc test	Control fish with no-stimulus versus control fish with stimulus	*p *< 10^−16^	Cohen's *d* (Hedges’ *g*) = 3.5
2*H*	Time showing orienting angle in ROI (%)	Tukey's post hoc test	cbln12::BoTx with no-stimulus versus cbln12::BoTx with stimulus	*p *= 1.3 × 10^−4^	Cohen's *d* (Hedges’ *g*) = 1.1
2*H*	Time showing orienting angle in ROI (%)	Tukey's post hoc test	Control fish with no-stimulus versus cbln12::BoTx with no-stimulus	*p *= 0.89	Cohen's *d* (Hedges’ *g*) = 0.23
2*H*	Time showing orienting angle in ROI (%)	Tukey's post hoc test	Control fish with stimulus versus cbln12::BoTx with stimulus	*p *= 0.0017	Cohen's *d* (Hedges’ *g*) = −1.0
2*I*	Time spent in ROI (%)	Two-way mixed ANOVA	BoTx × social stimulus interaction	*p *= 7.5 × 10^−4^; *F*_(1,18) _= 16	*η*^2 ^= 0.057
2*I*	Time spent in ROI (%)	Two-way mixed ANOVA	Main effect of BoTx	*p *= 0.080; *F*_(1,18) _= 3.4	*η*^2 ^= 0.039
2*I*	Time spent in ROI (%)	Two-way mixed ANOVA	Main effect of social stimulus	*p *= 6.6 × 10^−11^; *F*_(1. 18) _= 185	*η*^2 ^= 0.64
2*I*	Time spent in ROI (%)	Tukey's post hoc test	Control fish with no-stimulus versus control fish with stimulus	*p *< 10^−16^	Cohen's *d* (Hedges’ *g*) = 5.0
2*I*	Time spent in ROI (%)	Tukey's post hoc test	152B::BoTx with no-stimulus versus 152B::BoTx with stimulus	*p *= 2.8 × 10^−4^	Cohen's *d* (Hedges’ *g*) = 1.6
2*I*	Time spent in ROI (%)	Tukey's post hoc test	Control fish with no-stimulus versus 152B::BoTx with no-stimulus	*p *= 0.99	Cohen's *d* (Hedges’ *g*) = −0.15
2*I*	Time spent in ROI (%)	Tukey's post hoc test	Control fish with stimulus versus 152B::BoTx with stimulus	*p *= 0.0053	Cohen's *d* (Hedges’ *g*) = −1.5
2*J*	Time showing orienting angle in ROI (%)	Two-way mixed ANOVA	BoTx × social stimulus interaction	*p *= 0.39; *F*_(1,18) _= 0.79	*η*^2 ^= 0.0097
2*J*	Time showing orienting angle in ROI (%)	Two-way mixed ANOVA	Main effect of BoTx	*p *= 0.026; *F*_(1,18) _= 5.8	*η*^2 ^= 0.11
2*J*	Time showing orienting angle in ROI (%)	Two-way mixed ANOVA	Main effect of social stimulus	*p = *6.2 × 10^−5^; *F*_(1,18) _= 27	*η*^2 ^= 0.33
2*K*	Time spent in ROI (%)	Two-way mixed ANOVA	BoTx × social stimulus interaction	*p *= 0.56; *F*_(1,18) _= 0.36	*η*^2 ^= 0.0032
2*K*	Time spent in ROI (%)	Two-way mixed ANOVA	Main effect of BoTx	*p *= 0.0041; *F*_(1,18) _= 11	*η*^2 ^= 0.070
2*K*	Time spent in ROI (%)	Two-way mixed ANOVA	Main effect of social stimulus	*p *= 9.7 × 10^−8^; *F*_(1,18) _= 73	*η*^2 ^= 0.65
2*L*	Time showing orienting angle in ROI (%)	Two-way mixed ANOVA	BoTx × social stimulus interaction	*p *= 0.080; *F*_(1,18) _= 3.5	*η*^2 ^= 0.049
2*L*	Time showing orienting angle in ROI (%)	Two-way mixed ANOVA	Main effect of BoTx	*p *= 0.085; *F*_(1,18) _= 3.3	*η*^2 ^= 0.076
2*L*	Time showing orienting angle in ROI (%)	Two-way mixed ANOVA	Main effect of social stimulus	*p *= 0.0013; *F*_(1,18) _= 14	*η*^2 ^= 0.21
2*N*	Latency of peak correlation	Welch's *t* test	Control versus 152B::BoTx	*p *= 0.075; *t*_(22) _= 1.9	Cohen's *d* (Hedges’ *g*) = 0.58
2*P*	Latency of peak correlation	Welch's *t* test	Control versus cbln12::BoTx	*p *= 0.017; *t*_(25) _= 2.5	Cohen's *d* (Hedges’ *g*) = 0.70
2-1*A*	Swimming speed (cm/s)	Welch's *t* test	Control versus 152B::BoTx	*p *= 0.65; *t*_(36) _= 0.46	Cohen's *d* (Hedges’ *g*) = −0.14
2-1*B*	Turning frequency	Welch's *t* test	Control versus 152B::BoTx	*p *= 0.97; *t*_(37) _= 0.042	Cohen's *d* (Hedges’ *g*) = 0.013
2-1*C*	Swimming speed (cm/s)	Welch's *t* test	Control versus cbln12::BoTx	*p *= 0.75; *t*_(41) _= 0.32	Cohen's *d* (Hedges’ *g*) = 0.091
2-1*D*	Turning frequency	Welch's *t* test	Control versus cbln12::BoTx	*p *= 0.26; *t*_(27) _= 1.2	Cohen's *d* (Hedges’ *g*) = 0.32
3*C*	Time spent in ROI (%)	Two-way mixed ANOVA	BoTx × social stimulus interaction	*p *= 4.8 × 10^−5^; *F*_(1,38) _= 21	*η*^2 ^= 0.080
3*C*	Time spent in ROI (%)	Two-way mixed ANOVA	Main effect of BoTx	*p *= 0.14; *F*_(1,38) _= 2.2	*η*^2 ^= 0.018
3*C*	Time spent in ROI (%)	Two-way mixed ANOVA	Main effect of social stimulus	*p *= 2.8 × 10^−13^; *F*_(1,38) _= 119	*η*^2 ^= 0.45
3*C*	Time spent in ROI (%)	Tukey's post hoc test	Control fish with no-stimulus versus control fish with stimulus	*p *< 10^−16^	Cohen's *d* (Hedges’ *g*) = 3.9
3*C*	Time spent in ROI (%)	Tukey's post hoc test	aldoca:BoTx with no-stimulus versus aldoca:BoTx with stimulus	*p *= 0.0033	Cohen's *d* (Hedges’ *g*) = 0.9
3*C*	Time spent in ROI (%)	Tukey's post hoc test	Control fish with no-stimulus versus aldoca:BoTx with no-stimulus	*p *= 0.53	Cohen's *d* (Hedges’ *g*) = 0.62
3*C*	Time spent in ROI (%)	Tukey's post hoc test	Control fish with stimulus versus aldoca:BoTx with stimulus	*p *= 0.0015	Cohen's *d* (Hedges’ *g*) = −0.96
3*D*	Time showing orienting angle in ROI (%)	Two-way mixed ANOVA	BoTx × social stimulus interaction	*p *= 1.7 × 10^−4^; *F*_(1,38) _= 17	*η*^2 ^= 0.080
3*D*	Time showing orienting angle in ROI (%)	Two-way mixed ANOVA	Main effect of BoTx	*p *= 0.34; *F*_(1,38) _= 0.94	*η*^2 ^= 0.012
3*D*	Time showing orienting angle in ROI (%)	Two-way mixed ANOVA	Main effect of social stimulus	*p *= 1.2 × 10^−8^; *F*_(1,38) _= 52	*η*^2 ^= 0.24
3*D*	Time showing orienting angle in ROI (%)	Tukey's post hoc test	Control fish with no-stimulus versus control fish with stimulus	*p *= 7.0 × 10^−7^	Cohen's *d* (Hedges’ *g*) = 2.3
3*D*	Time showing orienting angle in ROI (%)	Tukey's post hoc test	aldoca:BoTx with no-stimulus versus aldoca:BoTx with stimulus	*p *= 0.40	Cohen's *d* (Hedges’ *g*) = 0.42
3*D*	Time showing orienting angle in ROI (%)	Tukey's post hoc test	Control fish with no-stimulus versus aldoca:BoTx with no-stimulus	*p *= 0.56	Cohen's *d* (Hedges’ *g*) = 0.56
3*D*	Time showing orienting angle in ROI (%)	Tukey's post hoc test	Control fish with stimulus versus aldoca:BoTx with stimulus	*p *= 0.021	Cohen's *d* (Hedges’ *g*) = −0.75
3*E*	Time spent in ROI (%)	Two-way mixed ANOVA	BoTx × social stimulus interaction	*p *= 0.064; *F*_(1,18) _= 3.9	*η*^2 ^= 0.038
3*E*	Time spent in ROI (%)	Two-way mixed ANOVA	Main effect of BoTx	*p *= 0.036; *F*_(1,18) _= 5.1	*η*^2 ^= 0.093
3*E*	Time spent in ROI (%)	Two-way mixed ANOVA	Main effect of social stimulus	*p *= 9.4 × 10^−6^; *F*_(1,18) _= 37	*η*^2 ^= 0.37
3*F*	Time showing orienting angle in ROI (%)	Two-way mixed ANOVA	BoTx × social stimulus interaction	*p *= 8.3 × 10^−4^; *F*_(1,18) _= 16	*η*^2 ^= 0.076
3*F*	Time showing orienting angle in ROI (%)	Two-way mixed ANOVA	Main effect of BoTx	*p *= 0.16 *F*_(1,18) _= 2.1	*η*^2 ^= 0.072
3*F*	Time showing orienting angle in ROI (%)	Two-way mixed ANOVA	Main effect of social stimulus	*p *= 1.9 × 10^−5^; *F*_(1,18) _= 33	*η*^2 ^= 0.16
3*F*	Time showing orienting angle in ROI (%)	Tukey's post hoc test	Control fish with no-stimulus versus control fish with stimulus	*p *= 0.0083	Cohen's *d* (Hedges’ *g*) = 1.3
3*F*	Time showing orienting angle in ROI (%)	Tukey's post hoc test	aldoca:BoTx with no-stimulus versus aldoca:BoTx with stimulus	*p *= 0.93	Cohen's *d* (Hedges’ *g*) = 0.31
3*F*	Time showing orienting angle in ROI (%)	Tukey's post hoc test	Control fish with no-stimulus versus aldoca:BoTx with no-stimulus	*p *= 1.0	Cohen's *d* (Hedges’ *g*) = −0.020
3*F*	Time showing orienting angle in ROI (%)	Tukey's post hoc test	Control fish with stimulus versus aldoca:BoTx with stimulus	*p *= 0.042	Cohen's *d* (Hedges’ *g*) = −1.1
3*H*	Latency of peak correlation	Welch's *t* test	Control versus aldoca:BoTx	*p *= 0.011; *t*_(24) _= 2.8	Cohen's *d* (Hedges’ *g*) = 0.86
3-1*A*	Swimming speed (cm/s)	Welch's *t* test	Control versus aldoca:BoTx	*p *= 0.043; *t*_(38) _= 2.1	Cohen's *d* (Hedges’ *g*) = −0.65
3-1*B*	Turning frequency	Welch's *t* test	Control versus aldoca:BoTx	*p *= 0.024; *t*_(31) _= 2.4	Cohen's *d* (Hedges’ *g*) = −0.74
4*B*	Time spent in ROI (%)	Two-way mixed ANOVA	Genotypes × social stimulus interaction	*p *= 0.039; *F*_(1,38) _= 4.6	*η*^2 ^= 0.020
4*B*	Time spent in ROI (%)	Two-way mixed ANOVA	Main effect of genotypes	*p *= 6.2 × 10^−4^; *F*_(1,38) _= 14	*η*^2 ^= 0.076
4*B*	Time spent in ROI (%)	Two-way mixed ANOVA	Main effect of social stimulus	*p *= 1.9 × 10^−13^; *F*_(1,38) _= 123	*η*^2 ^= 0.53
4*B*	Time spent in ROI (%)	Tukey's post hoc test	*reln^Δ7/+^* with no-stimulus versus *reln^Δ7/+^* with stimulus	*p *< 10^−16^	Cohen's *d* (Hedges’ *g*) = 2.8
4*B*	Time spent in ROI (%)	Tukey's post hoc test	*reln^Δ7/Δ7^* with no-stimulus versus *reln^Δ7/Δ7^* with stimulus	*p *= 5.0 × 10^−7^	Cohen's *d* (Hedges’ *g*) = 1.8
4*B*	Time spent in ROI (%)	Tukey's post hoc test	*reln^Δ7/+^* with no-stimulus versus *reln^Δ7/Δ7^* with no-stimulus	*p *= 0.53	Cohen's *d* (Hedges’ *g*) = −0.58
4*B*	Time spent in ROI (%)	Tukey's post hoc test	*reln^Δ7/+^* with stimulus versus *reln^Δ7/Δ7^* with stimulus	*p *= 4.0 × 10^−4^	Cohen's *d* (Hedges’ *g*) = −1.1
4*C*	Time showing orienting angle in ROI (%)	Two-way mixed ANOVA	Genotypes × social stimulus interaction	*p *= 0.038; *F*_(1,38) _= 4.6	*η*^2 ^= 0.036
4*C*	Time showing orienting angle in ROI (%)	Two-way mixed ANOVA	Main effect of genotypes	*p *= 0.11; *F*_(1,38) _= 2.7	*η*^2 ^= 0.025
4*C*	Time showing orienting angle in ROI (%)	Two-way mixed ANOVA	Main effect of social stimulus	*p *= 3.4 × 10^−7^; *F*_(1,38) _= 38	*η*^2 ^= 0.29
4*C*	Time showing orienting angle in ROI (%)	Tukey's post hoc test	*reln^Δ7/+^* with no-stimulus versus *reln^Δ7/+^* with stimulus	*p *= 1.9 × 10^−6^	Cohen's *d* (Hedges’ *g*) = 1.8
4*C*	Time showing orienting angle in ROI (%)	Tukey's post hoc test	*reln^Δ7/Δ7^* with no-stimulus versus *reln^Δ7/Δ7^* with stimulus	*p *= 0.041	Cohen's *d* (Hedges’ *g*) = 0.81
4*C*	Time showing orienting angle in ROI (%)	Tukey's post hoc test	*reln^Δ7/+^* with no-stimulus versus *reln^Δ7/Δ7^* with no-stimulus	*p *= 1.0	Cohen's *d* (Hedges’ *g*) = 0.078
4*C*	Time showing orienting angle in ROI (%)	Tukey's post hoc test	*reln^Δ7/+^* with stimulus versus *reln^Δ7/Δ7^* with stimulus	*p *= 0.045	Cohen's *d* (Hedges’ *g*) = −0.77
4*D*	Time spent in ROI (%)	Two-way mixed ANOVA	Genotypes × social stimulus interaction	*p *= 0.036; *F*_(1,18) _= 5.1	*η*^2 ^= 0.025
4*D*	Time spent in ROI (%)	Two-way mixed ANOVA	Main effect of genotypes	*p *= 0.032; *F*_(1,18) _= 5.4	*η*^2 ^= 0.046
4*D*	Time spent in ROI (%)	Two-way mixed ANOVA	Main effect of social stimulus	*p *= 5.7 × 10^−10^; *F*_(1,18) _= 142	*η*^2 ^= 0.69
4*D*	Time spent in ROI (%)	Tukey's post hoc test	*reln^Δ7/+^* with no-stimulus versus *reln^Δ7/+^* with stimulus	*p *< 10^−16^	Cohen's *d* (Hedges’ *g*) = 3.9
4*D*	Time spent in ROI (%)	Tukey's post hoc test	*reln^Δ7/Δ7^* with no-stimulus versus *reln^Δ7/Δ7^* with stimulus	*p *= 6.9 × 10^−6^	Cohen's *d* (Hedges’ *g*) = 2.4
4*D*	Time spent in ROI (%)	Tukey's post hoc test	*reln^Δ7/+^* with no-stimulus versus *reln^Δ7/Δ7^* with no-stimulus	*p *= 0.96	Cohen's *d* (Hedges’ *g*) = 0.24
4*D*	Time spent in ROI (%)	Tukey's post hoc test	*reln^Δ7/+^* with stimulus versus *reln^Δ7/Δ7^* with stimulus	*p *= 0.014	Cohen's *d* (Hedges’ *g*) = −1.23
4*E*	Time showing orienting angle in ROI (%)	Two-way mixed ANOVA	Genotypes × social stimulus interaction	*p *= 0.053; *F*_(1,18) _= 4.3	*η*^2 ^= 0.044
4*E*	Time showing orienting angle in ROI (%)	Two-way mixed ANOVA	Main effect of genotypes	*p *= 0.44; *F*_(1,18) _= 0.63	*η*^2 ^= 0.0089
4*E*	Time showing orienting angle in ROI (%)	Two-way mixed ANOVA	Main effect of social stimulus	*p *= 1.4 × 10^−6^; *F*_(1,18) _= 50	*η*^2 ^= 0.51
4*G*	Latency of peak correlation	Welch's *t* test	*reln^Δ7/+^* versus *reln^Δ7/Δ7^*	*p *= 0.069; *t*_(27) _= 1.9	Cohen's *d* (Hedges’ *g*) = 0.59
4-1*A*	Swimming speed (cm/s)	Welch's *t* test	*reln^Δ7/+^* versus *reln^Δ7/Δ7^*	*p *= 0.014 *t*_(33) _= 2.6	Cohen's *d* (Hedges’ *g*) = −0.80
4-1*B*	Turning frequency (/m)	Welch's *t* test	*reln^Δ7/+^* versus *reln^Δ7/Δ7^*	*p *= 0.43 *t*_(38) _= 0.80	Cohen's *d* (Hedges’ *g*) = −0.25
5*B*	Fold change of c-*fos* expression	Brunner–Munzel test	No-stimulus versus stimulus	*p *= 0.0033 Brunner–Munzel test statistic_(6.6) _= 4.5	Cliff's Delta = 0.84
5*C*	Fold change of *egr1* expression	Brunner–Munzel test	No-stimulus versus stimulus	*p *= 0.040 Brunner–Munzel test statistic_(8) _= 2.5	Cliff's Delta = 0.68
5-1*A*	Swimming speed (cm/s)	Paired *t* test	No-stimulus versus stimulus	*p *= 7.1 × 10^−7^; *t*_(19) _= 7.2	Cohen's *d* (Hedges’ *g*) = −2.0
5-1*B*	Turning frequency	Paired *t* test	No-stimulus versus stimulus	*p *= 0.59; *t*_(19) _= 0.54	Cohen's *d* (Hedges’ *g*) = 0.18

## Results

### Immediate establishment of orienting behavior in adult zebrafish

It was previously reported that late-stage larvae or adult zebrafish display stereotyped orienting behavior, in which a pair of zebrafish in two separate tanks approach each other, with one swimming at synchronized angles (orienting angles) with the partner (Stednitz et al., 2018; Stednitz and Washbourne, 2020). We analyzed the orienting behavior using a system similar to the one previously reported (Stednitz et al., 2018). Two (standard) tanks were placed across a divider made of an electrochromic film (Fig. 1*A*). A pair of fish was placed one by one in the tanks. After a 20 min acclimation, the fish were allowed to swim for 5 min with an opaque divider so that they could not see each other (no-stimulus condition), followed by 5 min with a transparent divider so that they could see each other (stimulus condition; Fig. 1*C*; Movie 1). We measured the degree to which fish exhibited the orienting behavior in a slightly different way than in the previous report (Stednitz et al., 2018). We determined the percentage of the time in which fish spent near the divider [25% of the tank divider side (ROI) in Fig. 1*A*] and the percentage of the time that fish looked at the partner fish at the head angle ±22.5–67.5° in the ROI (Fig. 1*B*). Whereas wild-type zebrafish stayed near the divider for 25 ± 1% (average ± SE) of the time under the no-stimulus condition, they stayed for 77 ± 5% of the time under the stimulus condition (Fig. 1*D*). Zebrafish displayed orienting head angles for 35 ± 2% of the time under the no-stimulus condition but 57 ± 3% under the stimulus condition (Fig. 1*E*). The data indicate that adult zebrafish stayed near the divider and showed orienting behavior for significantly longer periods under the stimulus condition than the no-stimulus condition (paired *t* test; *p *= 9.4 × 10^−10^; Cohen's *d *= 3.1 in Fig. 1*D*; paired *t* test; *p *= 1.1 × 10^−5^; Cohen's *d *= 1.9 in Fig. 1*E*), as reported (Stednitz et al., 2018). We also used a combination of standard and short tanks. We put test and stimulus fish in the standard and short tanks, respectively, and analyzed the test fish behavior (Fig. 1*F*,*G*). We confirmed that in this condition, wild-type adult zebrafish also showed the orienting behavior under the stimulus condition for longer periods than under the no-stimulus condition (time spent in the ROI, no-stimulus 26 ± 2% vs stimulus 79 ± 6%, paired *t* test; *p *= 4.1 × 10*^−5^*; Cohen's *d *= 3.3 in Fig. 1*F*; time showing orienting angle, no-stimulus 39 ± 3 vs stimulus 58 ± 5%, paired *t* test; *p *= 0.0023; Cohen's *d *= 1.5 in Fig. 1*G*). Previously studies have shown sex differences in shoaling behavior and orienting behavior in zebrafish (Ruhl et al., 2009; Way et al., 2015; Stednitz and Washbourne, 2020). We analyzed the differences in orienting behavior based on sex in AB wild-type fish. When measuring the time spent in the ROI under the no-stimulus condition, the percentages were as follows: test fish and stimulus fish combinations of female and female at 28 ± 4%, male and male at 34 ± 3%, female and male at 36 ± 4%, and male and female at 26 ± 4%. Under the stimulus condition, the percentages were as follows: female and female at 62 ± 6%, male and male at 78 ± 6%, female and male at 59 ± 12%, and male and female at 70 ± 11% (Extended Data Fig. 1-1*A*). When measuring the time showing orienting angle under no-stimulus conditions, the percentages were as follows: female and female at 20 ± 3%, male and male at 21 ± 2%, female and male at 16 ± 2%, and male and female at 22 ± 2%. Under the stimulus condition, the percentages were as follows: female and female at 36 ± 5%, male and male at 53 ± 6%, female and male at 32 ± 7%, and male and female at 41 ± 11% (Extended Data Fig. 1-1*B*). Since there were no significant differences based on the combinations of sex, we conducted the subsequent experiments with fish selected randomly with regard to sex. We examined whether previous experience seeing conspecifics is necessary to establish orienting behavior. We reared isolated adult zebrafish that had never seen other zebrafish before the behavior assay and analyzed their orienting behavior (Fig. 1*I*,*J*). The isolated zebrafish immediately established the orienting behavior under the stimulus condition as did zebrafish that grew with other zebrafish (time spent in the ROI, control 22 ± 3% vs isolate 24 ± 1% under no-stimulus condition, control 75 ± 5% vs isolate 78 ± 5% under stimulus condition in Fig. 1*H*; time showing orienting angle, control 40 ± 2% vs isolate 30 ± 1% under no-stimulus condition, control 54 ± 4% vs isolate 57 ± 3% under stimulus condition in Fig. 1*I*), indicating that previous social experience is not essential for the establishment of the orienting behavior. Furthermore, we examined whether zebrafish show the orienting behavior toward allospecific species. We used adult medaka with age as the test zebrafish as stimulus fish. Zebrafish orienting behavior toward medaka was much less than that toward zebrafish (time spent in the ROI, zebrafish 77 ± 6% vs medaka 45 ± 3%, paired *t* test; *p *= 0.0013; Cohen's *d *= 1.9 in Fig. 1*J*; showing orienting angle, zebrafish 58 ± 4% vs medaka 37 ± 3%, paired *t* test; *p *= 9.0 × 10^−4^; Cohen's *d *= 2.0 in Fig. 1*K*), supporting the previous notion that the orienting behavior in this assay is specific to conspecifics (Stednitz et al., 2018).

10.1523/ENEURO.0141-24.2024.f1-1 Figure 1-1 Sex difference on orienting behavior. Orienting behaviors were analyzed in combinations of test and stimulus fish: female and female (f > f), male and male (m > m), female and male (f > m), and male and female (m > f). Percentages of time spent in the ROI (***A***) and percentages of time that fish showed the orienting angles (***B)*** were measured. The time spent in the ROI and the time that fish showed the orienting angles were not significantly different across all possible sex pairings (female and female; *n *= 10, male and male; *n *= 10, female and male; *n *= 5, male and female; *n *= 5). Download Figure 1-1, TIF file.

**Movie 1.** Orienting behavior of wild-type zebrafish. A pair of wild-type fish swam for 5 min with an opaque divider preventing them from seeing each other (no-stimulus condition), followed by 5 min with a transparent divider allowing visibility (stimulus condition). The video has been shortened from 10 to 1 min.

### Inhibition of cerebellar GCs affects orienting behavior

**Figure 2.**
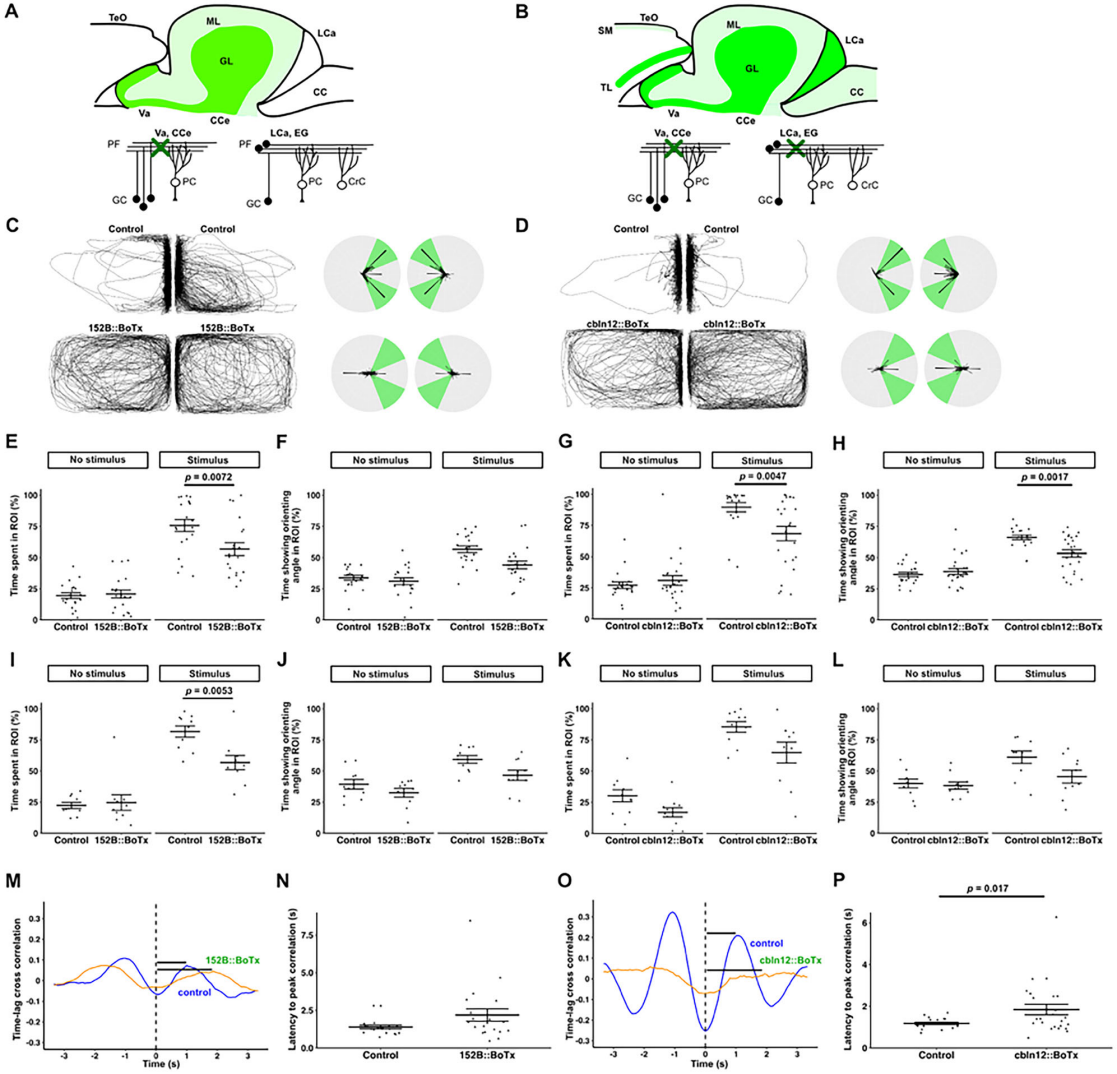
BoTx-mediated inhibition of GCs suppresses orienting behavior. Diagrams for the expression pattern of BoTx in adult transgenic zebrafish *gSA2AzGFF152B;Tg(UAS:BoTxBLC-GFP)* (***A***) and *Tg(cbln12:Gal4FF); Tg(UAS:BoTxBLC-GFP)* (***B***). CC, crista cerebellaris; CCe, corpus cerebelli; CrC, crest cell; EG, eminentia granularis; GL, granular layer; LCa, lobus caudalis cerebelli; ML, molecular layer; SM, stratum marginale; TeO, tectum opticum; TL, torus longitudinalis; Va, valvula cerebelli. Orienting behavior of 152B::BoTx, ***C***, ***E***, ***F***, ***I***, and ***J*** or cbln12::BoTx, ***D***, ***G***, ***H***, ***K***, and ***L***, which express BoTx in GCs. Sibling zebrafish that did not express BoTx were used as controls. Representative traces and polar histograms for 152B::BoTx and its control (***C***) and cbln12::BoTx and its control (***D***). ***C***–***H***, Orienting behavior in two large tanks. The same type of test fish (either BoTx-expressing or control fish) were placed in both tanks. ***I***–***L***, Orienting behavior toward wild-type control fish. A combination of standard and short tanks was used. The test fish was placed in the standard tank, while wild-type fish was placed in the short tank (for visual stimulus). The orienting behavior of the test fish in the one tank (***E***–***H***; large tank for ***I***–***K***, ***H***) was analyzed. Percentages of the time spent in the ROI (***E***, ***G***, ***I***, ***K***) and percentages of the time that fish showed the orienting angles in the ROI (***F***, ***H***, ***J***, ***L***) are indicated. 152B::BoTx spent less time in the ROI in the two large tank (*n *= 20 each in ***E***, ***F***) and large/small tank (*n *= 10 each in ***I***, ***J***) assays. cbln12::BoTx spent less time in the ROI and exhibited the orienting angles for a shorter duration than controls in the two large tank (control *n* = 20; cbln12::BoTx *n* = 24 in ***G***, ***H***) assays. Representative structures of time lag cross-correlation for 152B::BoTx and control fish (***M***) and cbln12::BoTx and control fish (***O***). Black bars indicate latency from time 0 to peak. Quantification of latency to peak correlation in 152B::BoTx and control fish (***N***) and cbln12::BoTx and control fish (***P***). Latency to peak correlation in cbln12::BoTx was significantly longer than that in control. See Extended Data Figure 2-1 for more details.

To examine the roles of cerebellar neural circuits in the orienting behavior, we used zebrafish Tg lines *gSA2AzGFF152B* and *Tg(cbln12:Gal4FF)*, which express a modified version of Gal4-VP16 (Gal4FF), in GCs (Takeuchi et al., 2015b; Matsuda et al., 2017; Koyama et al., 2021). We crossed them with *Tg(UAS:BoTxBLC-GFP)*, which expresses a fusion protein of the BoTx light chain B and green fluorescent protein (BoTx), which inhibits the release of neurotransmitters, in a Gal4-dependent manner (Sternberg et al., 2016; Lal et al., 2018), and reared them to adulthood (referred to as 152B::BoTx and cbln12::BoTx in Fig. 2). It was reported that 152B::BoTx expresses BoTx in GCs, mainly in the corpus cerebelli (rostromedial part of the cerebellum), while cbln12:BoTx expresses BoTx in GCs and a small population of telencephalic neurons (Fig. 2*A*,*B*; Koyama et al., 2021). Sibling zebrafish were used as control fish. We determined the percentage of the time that zebrafish spent near the divider (spent in the ROI; Fig. 2*C*,*D*,*E*,*G*) and the percentage of the time that zebrafish exhibited orienting angles (Fig. 2*C*,*D*,*F*,*H*). The 152B::BoTx fish started the orienting behavior under the stimulus condition as did control fish (Movie 2). However, they did so for shorter periods than control fish (time spent in the ROI, control 20 ± 2% vs 152B::BoTx 21 ± 3% under no-stimulus condition, control 76 ± 5% vs 152B::BoTx 57 ± 5% under stimulus condition, two-way mixed ANOVA, Tukey's post hoc test, control vs 152B::BoTx with stimulus condition; *p *= 0.0072; Cohen's *d *= −0.84 in Fig. 2*E*; time showing orienting angle, control 34 ± 2% vs 152B::BoTx 31 ± 3% under no-stimulus condition, control 57 ± 3% vs 152B::BoTx 44 ± 3% under stimulus condition in Fig. 2*F*; Movie 2). Similarly, cbln12::BoTx fish displayed the orienting behavior for shorter periods than control fish (time spent in the ROI, control 27 ± 3% vs cbln12::BoTx 31 ± 4% under no-stimulus condition, control 90 ± 4% vs cbln12::BoTx 68 ± 6% under stimulus condition in Fig. 2*G*; time showing orienting angle, control 36 ± 2% vs cbln12::BoTx 39 ± 2% under no-stimulus condition, control 66 ± 2% vs cbln12::BoTx 53 ± 3% under stimulus condition, two-way mixed ANOVA, Tukey's post hoc test, control vs cbln12::BoTx under stimulus condition; *p *= 0.0017; Cohen's *d *= −1.0 in Fig. 2*H*; Movie 3). To exclude the possibility that the stimulus Tg fish did not give sufficient cues to the test fish, we also used a combination of standard and short tanks and placed a wild-type AB fish as the stimulus fish in the short tank (Fig. 2*I*–*L*). Even in this condition, 152B::BoTx fish showed the orienting behavior for shorter periods than control fish (time spent in the ROI, control 22 ± 12% vs 152B::BoTx 25 ± 6% under no-stimulus condition, control 82 ± 5% vs 152B::BoTx 57 ± 6% under stimulus condition, two-way mixed ANOVA, Tukey's post hoc test, control vs 152B::BoTx under stimulus condition; *p *= 0.0053; Cohen's *d *= −1.5 in Fig. 2*I*; time showing orienting angle, control 39 ± 5% vs 152B::BoTx 33 ± 4% under no-stimulus condition, control 59 ± 3% vs 152B::BoTx 47 ± 4%; *p *= 0.019 under stimulus condition in Fig. 2*J*). When wild-type fish were used as stimulus fish, cbln12::BoTx fish showed the orienting angle in the ROI for shorter periods than control fish, but did not show significant differences in the time spent in the ROI and the time showing orienting angle, compared with control fish (time spent in the ROI, control 30 ± 10% vs cbln12::BoTx 17 ± 4% under no-stimulus condition, control 85 ± 4% vs cbln12::BoTx 65 ± 8% under stimulus condition in Fig. 2*K*; time showing orienting angle, control 40 ± 6% vs cbln12::BoTx 38 ± 3% under no-stimulus condition, control 61 ± 5% vs cbln12::BoTx 46 ± 5% under stimulus condition in Fig. 2*L*). These data indicate that inhibition of GCs shortened the orienting behavior, although there is some variation in the data. Consistent with the previous report (Koyama et al., 2021), the *ad libitum* swimming speed and turning frequency of 152B::BoTx and cbln12::BoTx fish under no-stimulus conditions were not significantly affected compared with control fish (swimming speed, control 4.0 ± 0.3 cm/s vs 152B::BoTx 3.8 ± 0.4 cm/s, Welch's *t* test; *p *= 0.65; Cohen's *d *= −0.14 in Extended Data Fig. 2-1*A*; turning frequency, control 52 ± 6 /m vs 152B::BoTx 52 ± 7 /m, Welch's *t* test; *p *= 0.97; Cohen's *d *= −0.013 in Extended Data Fig. 2-1*B*; swimming speed, control 4.4 ± 0.2 cm/s vs cbln12::BoTx 4.3 ± 0.3 cm/s, Welch's *t* test; *p *= 0.75; Cohen's *d *= −0.091 in Extended Data Fig. 2-1*C*; turning frequency, control 54 ± 3 /m vs cbln12::BoTx 66 ± 10 /m, Welch's *t* test *p *= 0.26, Cohen's *d *= −0.32 in Extended Data Fig. 2-1*D*). We analyzed time lag cross-correlation and measured latency to peak correlation (Fig. 2*M*–*P*), as reported previously (Stednitz and Washbourne, 2020). We found a significantly longer time lag in cross-correlation in cbln12::BoTx but not 152B::BoTx compared with control fish (control 1.4 ± 0.1 s vs 152B::BoTx 2.2 ± 0.4 s, Welch's *t* test; *p *= 0.075; Cohen's *d *= 0.58 in Fig. 2*N*; control 1.2 ± 0.1 s vs cbln12::BoTx 1.8 ± 0.2 s, Welch's *t* test; *p *= 0.017; Cohen's *d *= 0.70 in Fig. 2*P*). These data suggest that inhibiting GCs did not strongly affect *ad libitum* swimming but influenced the response to social cues and shortened orienting behavior.

10.1523/ENEURO.0141-24.2024.f2-1 Figure 2-1 Swimming behavior of GC-silenced zebrafish. Swimming speed of 152B::BoTx and control (***A***), cbln12::BoTx and control (***B***). Swimming speed under no-stimulus conditions was calculated. The swimming speed was not significantly different between 152B::BoTX and control (*n *= 20 each), and between cbln12::BoTx and control (*n *= 24 and *n *= 20, respectively). Turning frequency of 152B::BoTx and control (***C***), cbln12::BoTx and control (***D***). Turning frequency under no-stimulus conditions was calculated. The turning frequency was not significantly different between 152B::BoTx and control (**C**, *n *= 20 each) and between cbln12::BoTx and control (***D***, *n *= 24 and *n *= 20, respectively). Download Figure 2-1, TIF file.

**Movie 2.** Orienting behavior of a 152B::BoTx fish. A pair of control and 152B::BoTx fish swam for 5 min under no-stimulus condition, followed by 5 min under stimulus condition. The video displays the last 1 min of no-stimulus condition and the full 5 min of stimulus condition, compressed from a total of 6 to 1 min.

**Movie 3.** Orienting behavior of a cbln12::BoTx fish. A pair of control and cbln12::BoTx fish swam for 5 min under no-stimulus condition, followed by 5 min under stimulus condition. The video displays the last 1 min of no-stimulus condition and the full 5 min of stimulus condition, compressed from a total of 6 min to 1 min.

### Inhibition of PCs affects orienting behavior

**Figure 3.**
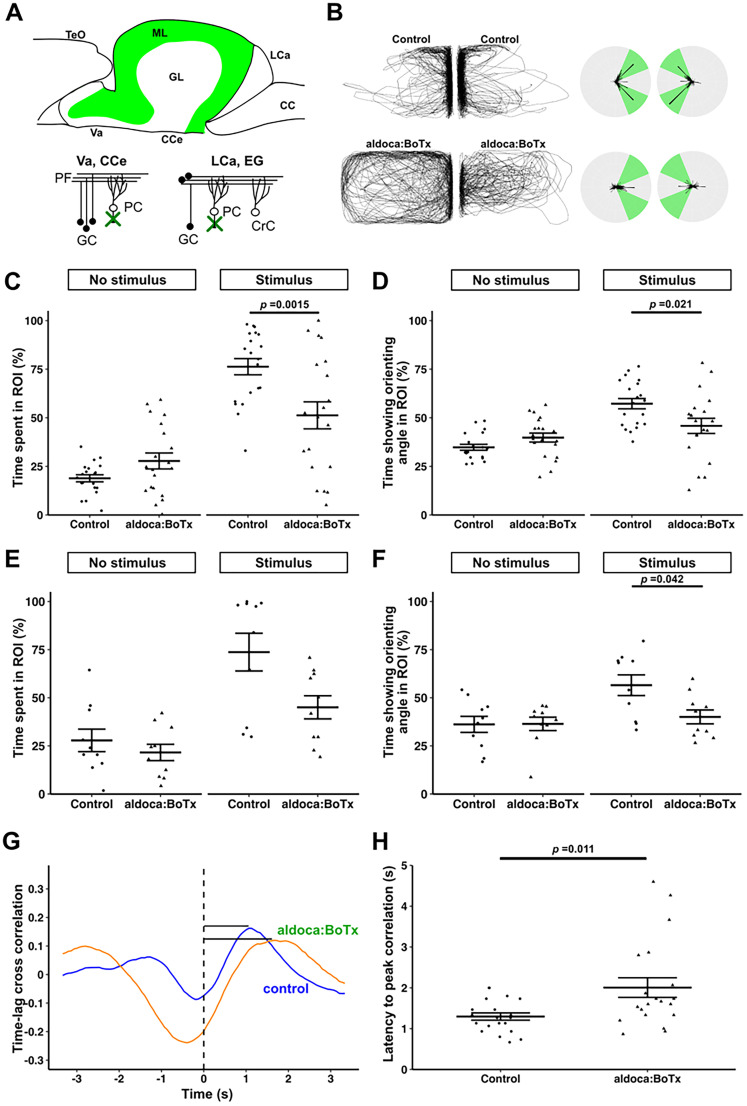
BoTx-mediated inhibition of PCs suppresses orienting behavior. Diagram for the expression pattern of BoTx in *Tg(aldoca:BoTxBLC-GFP)* (aldoca:BoTx) fish (***A***). aldoca::BoTx fish expresses BoTx in PCs. Orienting behavior of adult aldoca::BoTx fish. Sibling zebrafish that did not express BoTx were used as controls. ***B***–***D***, Orienting behavior in two large tanks. The same type of test fish was placed in both tanks. ***E***, ***F***, Orienting behavior toward wild-type control fish. A combination of large and small tanks was used. The test fish was placed in the large tank, while a wild-type fish was placed in the small tank (for visual stimulus). The orienting behavior of the test fish in the one tank (***C***, ***D***; large tank for ***E***, ***F***) was analyzed. Percentages of the time spent in the ROI (***C***, ***E***) and percentages of the time that fish showed the orienting angles in the ROI (***D***, ***F***) are indicated. aldoca:BoTx spent less time in the ROI and exhibited the orienting angles for a shorter duration than controls in both the two large tank (*n *= 20 in ***C***; *n *= 20 in ***D***) and large/small tank (*n *= 10 in ***E***; *n *= 10 in ***F***) assays. Representative structures of time lag cross-correlation for aldoca:BoTx and control fish (***G***). Black bars indicate latency from time 0 to peak. Quantification of latency to peak correlation in aldoca:BoTx and control fish (***H***). Latency to peak correlation in aldoca:BoTx was significantly longer than that in control. See Extended Data Figure 3-1 for more details

We next analyzed the role of PCs in orienting behavior by using *Tg(aldoca:BoTxBLC-GFP)* (referred to as aldoca:BoTx in Fig. 3), which expresses BoTx under the control of the enhancer and promoter of the PC-specific gene *aldolase* Ca (Fig. 3*A*; Koyama et al., 2021). Sibling fish that did not express BoTx were used as control fish. Similar to Tg fish expressing BoTx in GCs, aldoca:BoTx fish showed the orienting behavior under the stimulus condition but for shorter periods than the control (time spent in the ROI, control 19 ± 2% vs aldoca:BoTx 28 ± 4% under no-stimulus condition, control 76 ± 4% vs aldoca:BoTx 51 ± 7% under stimulus condition, two-way mixed ANOVA, Tukey's post hoc test, control vs aldoca:BoTx under stimulus condition; *p *= 0.0015; Cohen's *d *= −0.96 in Fig. 3*C*; time showing orienting angle, control 35 ± 2% vs aldoca:BoTx 40 ± 2% under no-stimulus condition, control 57 ± 3% vs aldoca:BoTx 46 ± 4% under stimulus condition, two-way mixed ANOVA, Tukey's post hoc test, control vs aldoca:BoTx under stimulus condition; *p *= 0.021; Cohen's *d *= −0.75 in Fig. 3*D*; Movie 4). When wild-type fish were used as stimulus fish, aldoca:BoTx fish also showed the orienting behavior for shorter periods than the control (time spent in the ROI, control 28 ± 0.3% vs aldoca:BoTx 22 ± 4% under no-stimulus, control 74 ± 10% vs aldoca:BoTx 45 ± 6% under stimulus in Fig. 3*E*; time showing orienting angle, control 36 ± 2% vs aldoca:BoTx 36 ± 3% under no-stimulus, control 57 ± 5% vs aldoca:BoTx 40 ± 4% under stimulus condition, two-way mixed ANOVA, Tukey's post hoc test, control vs aldoca:BoTx under stimulus condition; *p *= 0.042; Cohen's *d *= −1.1 in Fig. 3*F*). The data indicate that inhibition of PCs shortened the orienting behavior. The BoTx-mediated PC inhibition did not affect the *ad libitum* swimming speed in the previous report (Koyama et al., 2021). However, the swimming speed of aldoca:BoTx fish was lower than that of the control (control 4.2 ± 0.3 cm/s vs aldoca:BoTx 3.4 ± 0.3 cm/s, Welch's *t* test; *t*_(38) _= 2.1; *p *= 0.043; Cohen's *d *= −0.65 in Extended Data Fig. 4-1*A*). Since the tanks of different shapes were used in this assay, the difference in the swimming speed may be attributed to the shape of the tanks. The turning frequency of aldoca:BoTx fish was also lower than that of the control fish (control 65 ± 7 /m vs aldoca:BoTx 45 ± 4 /m, Welch's *t* test; *t*_(31) _= 2.4; *p *= 0.024; Cohen's *d *= −0.74 in Extended Data Fig. 4-1*B*). We analyzed time lag cross-correlation and measured latency to peak correlation (Fig. 3*G*,*H*). We found a significantly longer time lag in cross-correlation in aldoca:BoTx fish compared with control fish (control 1.3 ± 0.1 s vs aldoca:BoTx 2.0 ± 0.2 s, Welch's *t* test; *p *= 0.011; Cohen's *d *= 0.86 in Fig. 3*H*). The data indicate that the inhibition of PCs affected both orienting behavior and *ad libitum* swimming.

10.1523/ENEURO.0141-24.2024.f3-1 Figure 3-1 Swimming behavior of PC-silenced zebrafish. Swimming speed of aldoca:BoTx and control (***A***). Swimming speed under no-stimulus conditions was calculated. The swimming speed of aldoca:BoTx was lower than that of the control (***A***, *n *= 20 each). Turning frequency of aldoca:BoTx and control (***B***). Turning frequency under no-stimulus conditions was calculated. The turning frequency of aldoca:BoTx was lower than that of the control (***B***, *n *= 20 each). Download Figure 3-1, TIF file.

**Movie 4.** Orienting behavior of an aldoca:BoTx fish. A pair of control and aldoca:BoTx fish swam for 5 min under no-stimulus condition, followed by 5 min under stimulus condition. The video displays the last 1 min of no-stimulus condition and the full 5 min of stimulus condition, compressed from a total of 6 to 1 min.

### *Reln* mutants affects orienting behavior

**Figure 4.**
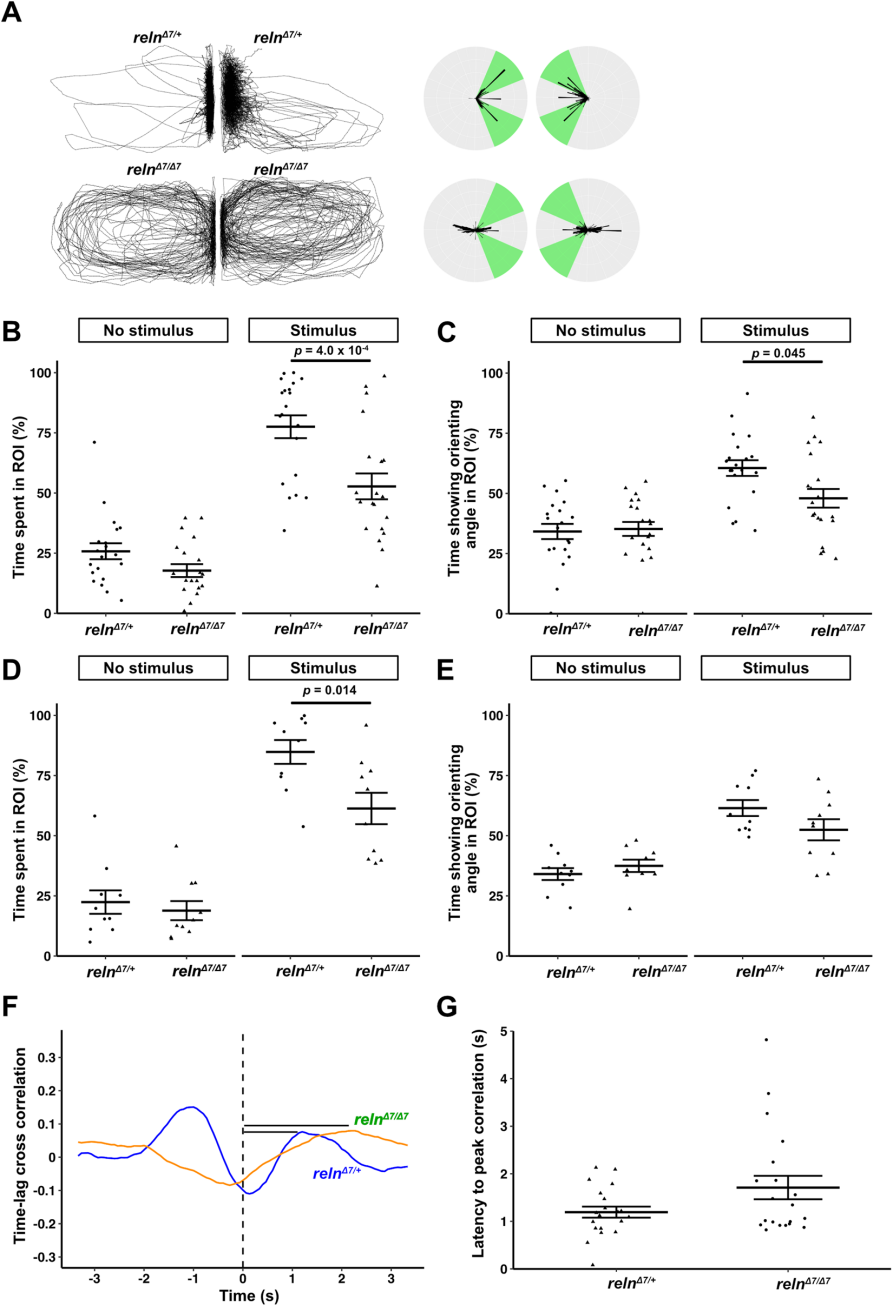
*reln* mutants show defective orienting behavior. Orienting behavior of adult *reln* mutant homozygous fish (*reln^Δ7/Δ7^*) and control heterozygous fish (*reln^Δ7/+^*). ***A***–***C***, Orienting behavior in two large tanks. The same genotype of fish was placed in both tanks. ***D***, ***E***, Orienting behavior toward wild-type control fish. A combination of large and small tanks was used. The test fish was placed in the large tank, while a wild-type fish was placed in the small tank. Percentages of the time spent in the ROI (***B***, ***D***) and percentages of the time that fish showed the orienting angle (***C***, ***E***) are indicated. *reln^Δ7/Δ7^* spent less time in the ROI and exhibited the orienting behavior for a shorter duration than controls in both the two large tank (*n *= 20 each in ***B***, ***C***) and large/small tank (*n *= 10 each in ***D***, ***E***) assays. Representative structures of time lag cross-correlation for *reln^Δ7/+^* and *reln^Δ7/Δ7^* fish (***F***). Black bars indicate latency from time 0 to peak. Quantification of latency to peak correlation in *reln^Δ7/Δ7^* and *reln^Δ7/+^* fish (***G***). See Extended Data Figure 4-1 for more details.

We then examined a zebrafish mutant of *reln*, which is required for the proper positioning of neurons in the cerebellum and the cerebellum-like structure in zebrafish (Nimura et al., 2019). The *reln* mutant allele *reln^Δ7^* used in this study is considered to be a null allele (Nimura et al., 2019). We examined the orienting behavior of homozygous fish (*reln^Δ7/Δ7^*) and compared it to heterozygous control fish (*reln^Δ7/+^*). *reln^Δ7/Δ7^* fish showed the orienting behavior under the stimulus condition but for shorter periods than *reln^Δ7/+^* (time spent in ROI, *reln^Δ7/+^* 26 ± 3% vs *reln^Δ7/Δ7^* 18 ± 3% under no-stimulus condition, *reln^Δ7/+^* 78 ± 5% vs *reln^Δ7/Δ7^* 53 ± 5% under stimulus condition, two-way mixed ANOVA, Tukey's post hoc test, *reln^Δ7/+^* vs *reln^Δ7/Δ7^* under stimulus condition; *p *= 4.0 × 10^−4^; Cohen's *d *= −1.1 in Fig. 4*B*; time showing orienting angle *reln^Δ7/+^* 34 ± 3% vs *reln^Δ7/Δ7^* 35 ± 3% under no-stimulus condition, *reln^Δ7/+^* 61 ± 3% vs *reln^Δ7/Δ7^* 48 ± 4% under stimulus condition, two-way mixed ANOVA, Tukey's post hoc test, *reln^Δ7/+^* vs *reln^Δ7/Δ7^* under stimulus condition; *p *= 0.045; Cohen's *d *= −0.77 in Fig. 4*C*; Movie 5). When wild-type fish were used as stimulus fish, *reln^Δ7/Δ7^* fish also stayed in the ROI for shorter periods than *reln^Δ7/+^* (*reln^Δ7/+^* 22 ± 14% vs *reln^Δ7/Δ7^* 19 ± 4% under no-stimulus condition, *reln^Δ7/+^* 85 ± 5% vs *reln^Δ7/Δ7^* 61 ± 7% under stimulus condition, two-way mixed ANOVA, Tukey's post hoc test; *reln^Δ7/+^* vs *reln^Δ7/Δ7^* under stimulus condition; *p *= 0.014; Cohen's *d *= −1.2 in Fig. 4*D*), but did not show a significant difference in the time spent showing orienting angle compared with *reln^Δ7/+^* (*reln^Δ7/+^* 34 ± 7% vs *reln^Δ7/Δ7^* 37 ± 3% under no-stimulus condition, *reln^Δ7/+^* 61 ± 3% vs *reln^Δ7/Δ7^* 52 ± 4% under stimulus condition in Fig. 4*E*). These data indicate that *reln* deficiency shortened the orienting behavior. The swimming speed of *reln^Δ7/Δ7^* fish was lower than that of *reln^Δ7/+^* fish (*reln^Δ7/+^* 3.6 ± 0.2 cm/s vs *reln^Δ7/Δ7^* 2.7 ± 0.3 cm/s, Welch's *t* test; *p *= 0.014; Cohen's *d *= −0.80 in Extended Data Fig. 4-1*A*). The turning frequency of *reln^Δ7/Δ7^* fish under the no-stimulus condition was not significantly affected compared with control fish (*reln^Δ7/+^* 59 ± 7 /m vs *reln^Δ7/Δ7^* 51 ± 7 /m, Welch's *t* test; *p *= 0.43; Cohen's *d *= −0.25 in Extended Data Fig. 4-1*B*). We analyzed time lag cross-correlation and measured latency to peak correlation (Fig. 4*F*,*G*). We did not find a significant difference in latency to peak correlation between *reln^Δ7/Δ7^* fish compared with *reln^Δ7/+^* fish (*reln^Δ7/+^* 1.2 ± 0.1 s vs *reln^Δ7/Δ7^* 1.7 ± 0.2 s, Welch's *t* test; *p *= 0.069; Cohen's *d *= 0.59 in Fig. 4*G*). The data indicate that *reln* mutant fish showed abnormalities in both orienting behavior and swimming speed.

10.1523/ENEURO.0141-24.2024.f4-1 Figure 4-1 Swimming behavior of *reln* mutant zebrafish. Swimming speed of *reln^Δ7/Δ7^* and *reln^Δ7/+^* (***A***). Swimming speed under no-stimulus conditions was calculated. The swimming speed of *reln^Δ7/Δ7^* was lower than *reln^Δ7/+^* (***A***, *n *= 20 each). Turning frequency of *reln^Δ7/Δ7^* and *reln^Δ7/+^* (***B***). Turning frequency under no-stimulus conditions was calculated. The turning frequency was not significantly different between *reln^Δ7/Δ7^* and *reln^Δ7/+^*(***B***, *n *= 20 each). Download Figure 4-1, TIF file.

**Movie 5.** Orienting behavior of a *reln^Δ7/Δ7^* fish. A pair of control and *reln^Δ7/Δ7^* fish swam for 5 min under no-stimulus condition, followed by 5 min under stimulus condition. The video displays the last 1 min of no-stimulus condition and the full 5 min of stimulus condition, compressed from a total of 6 to 1 min.

### The cerebellum is activated in orienting behavior

**Figure 5.**
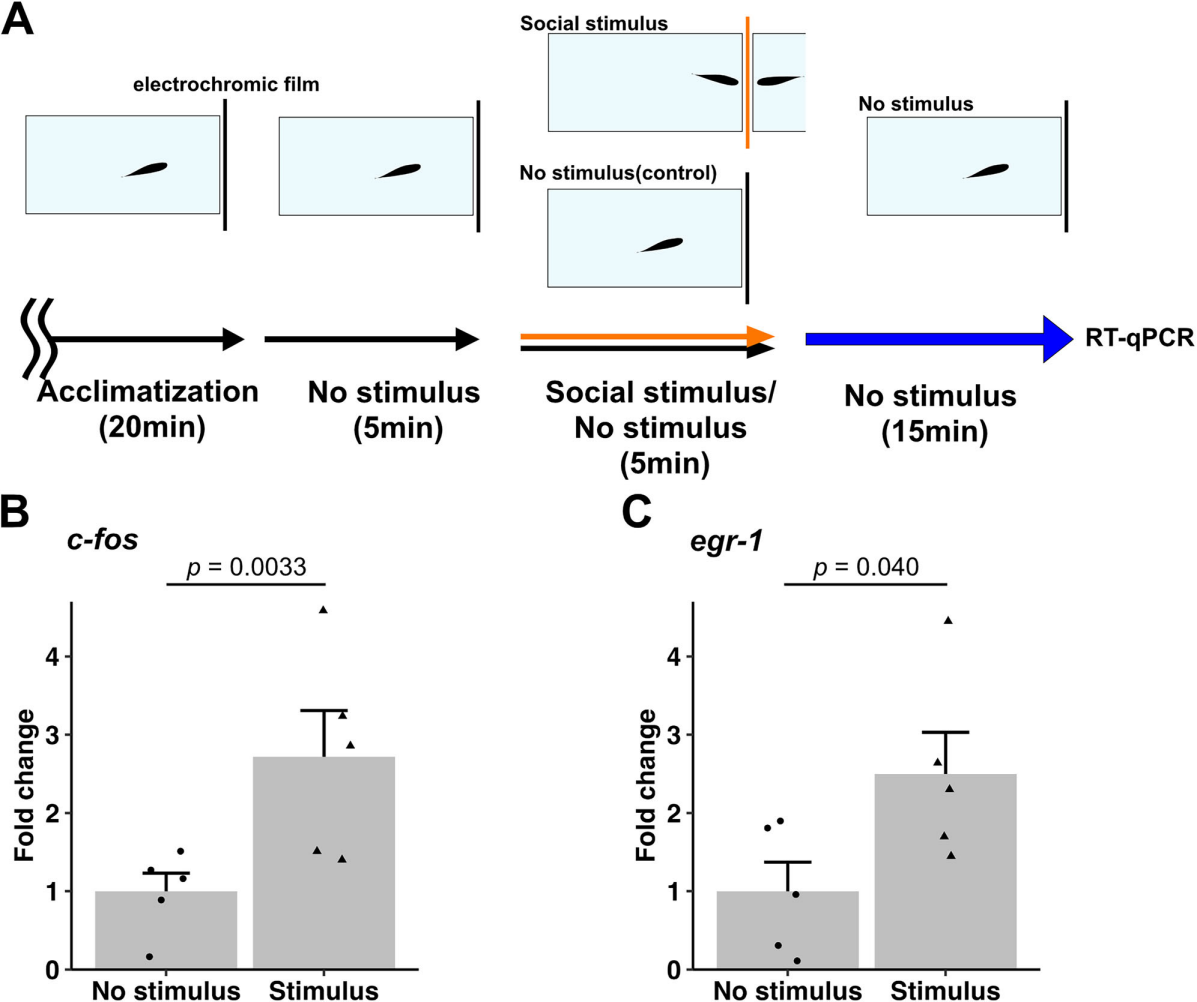
The cerebellum is activated during orienting behavior. ***A***, Detection of neuronal activity during orienting behavior. After 20 min of acclimatization, zebrafish were allowed to swim for 5 min under no-stimulus condition, followed by 5 min of orienting behavior assays under no-stimulus or stimulus conditions. For RT-qPCR, zebrafish were further kept for 15 min under no-stimulus condition, and then RNA from the cerebellum was isolated. ***B***, ***C***, c-*fos* (***B***) and *egr1* (***C***) expressions detected by RT-qPCR. c-*fos* and *egr1* transcripts were amplified from cDNAs from the cerebellum of zebrafish under no-stimulus or stimulus conditions. Data are shown as ratios to expression in fish under no-stimulus conditions. Expressions of c-*fos* and *egr1* mRNA significantly increased in the cerebellum of zebrafish that exhibited orienting behavior under stimulus conditions, compared with no-stimulus conditions (***B***, c-*fos n *= 5, ***C***; *egr1 n *= 5). See Extended Data Figure 5-1 for more details.

Furthermore, we analyzed whether the cerebellum is activated in the orienting behavior. We examined the expression of immediate early genes c-*fos* and *egr1* mRNA as a readout of neural activation (Lau et al., 2011; Teles et al., 2015; Wakisaka et al., 2017; Ariyasiri et al., 2021; Hodge et al., 1998; Kidambi et al., 2009). Zebrafish brains were harvested 5 min after the orienting behavior assays to detect c-*fos* and *egr1* mRNA expression by RT-qPCR (Fig. 5*A*). The expression of c-*fos* and *egr1* in the cerebellum was significantly increased after the orienting behavior the under stimulus condition, compared with the no-stimulus condition (c-*fos*, 2.7 ± 0.6-fold; Brunner–Munzel test statistic_(6.6) _= 4.5; *p *= 0.0033; Cliff's Delta = 0.84 in Fig. 5*B*; *egr1*, 2.5 ± 0.5-fold; Brunner–Munzel test statistic_(8.0) _= 2.5; *p *= 0.040; Cliff's Delta = 0.68 in Fig. *5C*). The cerebellum is involved in motor control, and cerebellar neural activity may also be related to locomotion. Therefore, we analyzed the swimming speed and turning frequency of fish under the social stimulus and no-stimulus conditions. The results showed that swimming speed significantly decreased under the stimulus condition (no-stimulus 5.1 ± 0.2 s vs stimulus 3.1 ± 0.3 s, paired *t* test; *p *= 7.1 × 10^−7^; Cohen's *d *= −2.0 in Extended Data Fig. 5-1*A*), and there was a slight increase in turning frequency, but no statistically significant differences were observed (no-stimulus 61 ± 4 /m vs stimulus 65 ± 7 /m, paired *t* test; *p *= 0.59; Cohen's *d *= 0.18 in Extended Data Fig. 5-1*B*). The data suggest that the cerebellum was activated at some point during the orienting behavior and this activity was not correlated with locomotion.

10.1523/ENEURO.0141-24.2024.f5-1 Figure 5-1 Swimming behavior of adult zebrafish. Swimming speed of adult zebrafish under no-stimulus or social stimulus conditions (***A***). Turning frequency of adult zebrafish under no-stimulus or social stimulus conditions (***B***). The swimming speed significantly decreased in the fish that exhibited orienting behavior under stimulus conditions, compared to no-stimulus conditions (***A***, *n *= 20). Turning frequency did not change in the fish that showed orienting behavior under stimulus conditions, compared to no-stimulus conditions (***B***, *n *= 20). Download Figure 5-1, TIF file.

## Discussion

### Orienting behavior

It was previously reported that zebrafish of different genetic backgrounds (ABxTU vs WIK) exhibited the orienting behavior (Stednitz et al., 2018). In this study, we found that zebrafish did not show the orienting behavior much toward medaka (Fig. 1). Although it is not clear whether the difference in appearance between zebrafish and medaka, or the difference in the way they swim, caused the defective orienting behavior, medaka could not provide zebrafish with enough social cues to induce the orienting behavior and vice versa. The data support the idea that the orienting behavior is specific to conspecific species. Further analysis, such as orienting behavior with virtual fish with various elemental changes, may be useful to identify the necessary elements for the orientation behavior. Furthermore, we found that zebrafish that had never seen other zebrafish (i.e., had no experience with sociality) immediately exhibited the orienting behavior (Fig. 1), indicating that previous social experiences are not necessary for this behavior. Thus, the orienting behavior does not seem to depend on repetitive learning processes. Social preference for conspecifics of zebrafish may be an innate behavior. Alternatively, similar to birds’ imprinting, where brief exposure to particular types of objects during the early years of life attracts them to the object (Hess, 1964), zebrafish were able to quickly learn how to respond to social cues. A previous study has demonstrated that social preference decreases in 3-week-old zebrafish when they are raised entirely without social interaction or isolated for 48 h before the behavioral assay (Tunbak et al., 2020). The age of zebrafish and the shape of the experimental tank used in our study, which differ from those in the previous study, may contribute to the difference in results. In our experimental system, we have clearly shown that adult zebrafish exhibit orienting behavior without any existence of social interaction. The zebrafish cerebellar neural circuitry is involved not only in orienting behavior but also in classical fear conditioning and active avoidance conditioning (Matsuda et al., 2017; Koyama et al., 2021). Classical and active avoidance conditioning require repetitive training involving pairs of unconditioned and conditioned stimuli, whereas the orienting behavior does not require such repetition. It is likely that orienting behavior is controlled by a different mechanism than classical and active avoidance conditioning.

### Cerebellar neural circuits are involved in orienting behavior

BoTx-mediated inhibition of GCs (152B::BoTx and cbln12::BoTx) or PCs (aldoca:BoTx) shortened the time that zebrafish displayed the orienting behavior (Figs. 2, 3). A zebrafish mutant of *reln*, which is required for the positioning of neurons and/or glial cells in the cerebellum (Nimura et al., 2019), displayed shortened orienting behavior (Fig. 4). The Tg fish used in this analysis do not express BoTx in the neural circuits involved in visual information processing. However, it cannot be completely ruled out that they lack visual abnormalities. Furthermore, abnormalities in visual circuits have been reported in *reln* mutant fish (Di Donato et al., 2018), and we cannot entirely exclude their involvement. However, the Tg fish and the *reln* mutant fish swam toward the divider and stayed near it to some extent compared with no-stimulus condition (Figs. 2*C*, 3*B*, 4*A*; Movies 2–5). Therefore, it is likely that there were no serious abnormalities in the visual information pathways of these fish. The data suggest that cerebellar neural circuits are involved in the orienting behavior.

These cerebellum-deficient zebrafish had different properties. First, both 152B::BoTx and cbln12::BoTx fish expressed BoTx in the GCs of the corpus cerebelli, but 152B::BoTx and cbln12::BoTx zebrafish also expressed BoTx in the telencephalon and GCs in the caudal lobe of the cerebellum, respectively (Koyama et al., 2021). Considering that 152B::BoTx and cbln12::BoTx fish exhibited similar abnormalities in the orienting behavior, GCs in the corpus cerebelli may be responsible for the orienting behavior. Neither 152B::BoTx nor cbln12::BoTx fish showed abnormalities in swimming speed or turning frequency. However, aldoca::BoTx fish exhibited shortened orienting behavior as well as decreased swimming speed and turning frequency. This is consistent with reports that aldoca::BoTx fish generate erratic forms of body displacement under certain conditions (Chang et al., 2020). Given that the cerebellum is involved in motor coordination and motor learning, it is possible that 152B::BoTx and cbln12::BoTx fish also have subtle abnormalities in motor control. The differences between aldoca::BoTx and 152B::BoTx or cbln12::BoTx may reflect the extent of the inhibitory effects on cerebellar neural circuits rather than differences in the functions of PCs and GCs. Orienting behavior, where a fish moves toward and responds to its partner, depends on motor control, making it difficult to easily separate the effects of cerebellar inhibition on motor control and social behavior based on the behavioral analysis of cerebellum-deficient zebrafish. Nevertheless, this study indicates that cerebellar neural circuits are involved in both motor control and social behavior.

Reln is not only involved in the positioning of neurons and glial cells in the cerebellum but also in the cerebellum-like structure in the mesencephalon in zebrafish (Nimura et al., 2019). Reln is also involved in the targeting of retinal ganglion cells to the mesencephalic tectum in zebrafish (Di Donato et al., 2018). Reln and its signaling molecules are expressed in various regions of the brain in zebrafish (Costagli et al., 2002; Imai et al., 2012). Considering the variety of roles of Reln signaling in brain development in mammals (Rice and Curran, 2001), abnormal development in brain regions other than the cerebellum may also contribute to defects in the orienting behavior. *reln* mutant zebrafish reportedly showed an abnormal preference for social novelty, preferring to approach zebrafish that they see for the first time (Dalla Vecchia et al., 2019). In this study, *reln* mutant zebrafish showed abnormal orienting behavior (Fig. 4). Therefore, Reln is involved in multiple processes of social behaviors. Alternatively, the same neural circuit mechanisms that rely on Reln signaling may control both orienting and social novelty preference behaviors. It is not clear whether Reln signaling in the cerebellum is involved in these social behaviors and whether the social novelty preference and the orienting behavior are controlled by the same neural circuit mechanisms. Specific inhibition of *reln* in the cerebellum may answer this question. Allelic variants of the human *Reln* gene are associated with certain types of ASD (Lammert et al., 2017; Sanchez-Sanchez et al., 2018; Hernandez-Garcia et al., 2020; Nawa et al., 2020; Scala et al., 2022). Further studies of zebrafish mutants of Reln and Reln signaling molecules in the orienting behavior may reveal the relationship between abnormality in Reln signaling and ASD.

The zebrafish models used in this study all exhibited abnormalities in cerebellar development and function from their early stages. This suggests that they may have developed compensatory neural circuits to mitigate the impacts of these abnormalities on their social behavior, potentially resulting in milder abnormalities than what would be observed with an acute loss of cerebellar functions. Since the depletion of PCs in adult zebrafish led to more severe abnormalities in *ad libitum* swimming (Koyama et al., 2021), we were unable to determine the role of the cerebellar circuits in the absence of these compensatory pathways. The use of optogenetic and/or chemogenetic approaches to inhibit cerebellar neural circuits in adult zebrafish will help to clarify this issue.

### Neural circuit structure for orienting behavior

Although the cerebellar neural circuits are involved in the orienting behavior, it is unclear how they control it. We found increased expression of the immediate early genes in the cerebellum after the orienting behavior (Fig. 5), suggesting that cerebellar neurons are activated by the orienting behavior. However, since zebrafish were actively moving during the orienting behavior, it is currently difficult to distinguish between activities that control the orienting behavior and those that control locomotion. Functional imaging of the cerebellum during the orienting behavior may clarify specific circuit elements involved in the orienting behavior and what information is linked to cerebellar activity.

It has been reported that cholinergic neurons expressing *lhx8a*, which are present in the ventral telencephalon equivalent to the lateral septum in mammals, and dopaminergic neurons in the preoptic region are involved in the orienting behavior (Stednitz et al., 2018; Tallafuss et al., 2022). Furthermore, abnormalities in the PFC, amygdala, and hippocampus, in addition to the cerebellum, are implicated in ASD (Bauman and Kemper, 2005). Therefore, the zebrafish-equivalent brain regions of these areas may also be involved in the orienting behavior. Visual information is crucial for the orienting behavior, and the pretectum, tectum, and dorsal telencephalon regions that receive visual information should be involved. It will be necessary to examine how these regions are activated during the orienting behavior. By examining the neural circuit connections between these areas, and by investigating the effects on the activity of other areas and on the orienting behavior when these regions are activated or inhibited, the neural circuit mechanisms controlling social behavior, including the cerebellum, will be elucidated.

### How do the cerebellar neural circuits control orienting behavior?

During the orienting behavior, a zebrafish responds to visual social cues from its partner and displays the same behavior with a short time lag (∼1 s; Stednitz et al., 2018; Stednitz and Washbourne, 2020). Thus, zebrafish need to predict and adapt to their partner's movements. The cerebellar neural circuits are known to be involved in prediction and adaptation for motor control. Masao Ito proposed a theoretical concept for cerebellar function in which the cerebellum provides the brain with a way to generate internal models for smooth motor actions (Ito, 2006). Internal models are neural representations that reproduce the dynamic properties of body parts. Animals perform movements as predicted by the internal model. If the prediction is not correct, the internal model is revised, and the animal performs a more adapted behavior smoothly and efficiently. Masao Ito extended this idea to explain the cerebellar control of mental activities (Ito, 2008). It has been proposed that deficits in prediction and adaptation via the internal models created by cerebellar neural circuits may be involved in ASD (Kelly et al., 2021). Zebrafish cerebellar circuits may also function in the establishment of internal models for social behavior. The cerebellar neural circuit internal model is thought to be used for motor control and motor learning (Ito, 2008), and through this internal model, the same mechanism may be involved in both motor control and social behavior. In addition to precise synchronous swimming, the orienting behavior is characterized by long duration (from 10 min to an hour). Social reward mechanisms should be involved. The cerebello-VTA connectivity, which activates the dopaminergic system, has been also shown to be involved in reward signals for social behavior in mice (Carta et al., 2019). Zebrafish may have similar connectivity, which may involve the dopaminergic system necessary for long-lasting orienting behavior.

In summary, cerebellar neural circuits are required for the proper performance of the orienting behavior, which serves as a model of human social behavior. Studies using zebrafish models will shed light on the mechanisms by which cerebellar circuits control social behaviors and the types of cerebellar abnormalities that lead to the manifestation of ASD.

## Synthesis

Reviewing Editor: David Schoppik, New York University Decisions are customarily a result of the Reviewing Editor and the peer reviewers coming together and discussing their recommendations until a consensus is reached. When revisions are invited, a fact-based synthesis statement explaining their decision and outlining what is needed to prepare a revision will be listed below. The following reviewer(s) agreed to reveal their identity: Sarah Stednitz, Jovin Jacobs.

1. Although the authors test social behavior in a purely visual assay, they do not discuss the potential visual deficits in the transgenic and mutant lines used here. In particular, it is known that reelin is required for normal synapse formation in the zebrafish visual system (see Donato et al, 2018, cited in the discussion on pg 20). The possibility that these phenotypes are a result of visual defects that are not unique to social behavior should be discussed. Additional analysis of the existing data, by showing that test fish approach the divider even if they are on the opposite end of the tank when it becomes transparent, would also support the idea they can at least perceive the stimulus. Otherwise, the authors could re-express interpretation of their results with this caveat in mind.

2. Plotting the raw data of orienting angles as polar histograms would aid in the interpretation of results. This is shown as a diagram in Figure 1B, but these data are not plotted in subsequent figures. Representative examples from each genotype tested would support the accompanying scatter plots. Similarly, it would be helpful to see the xy coordinates plotted to evaluate if swimming behavior is otherwise unperturbed in the test animals.

3. The statistical comparisons used here are unclear. If the behavior of the fish during the social stimulus are being compared to the behavior of fish in the pre-stimulus period (eg, the same animals), then it is more appropriate to use a repeated measures test with a within-subjects design. Please clarify what comparisons are being made and use the appropriate repeated measures statistics if necessary.

4. The increased expression of egr1 and c-fos in the social condition could be related to an increase in swimming rate, as mentioned on pg 21. If it's possible to match the individual behavioral data to these samples, comparing expression levels of individual animals to the locomotor activity could clarify this point, and potentially strengthen the author's argument that it is specific to social interaction.

5. The authors suggest that the timing of responses to social cues may be perturbed in aldoca:BoTx fish, which is an interesting point (pg 19). If both the stimulus and target animals have been tracked, it would be possible to calculate a time-lag cross correlation between each animal's orientation. This could reveal if the deficits are related to slower response times in the test fish.

6. The cerebellum's involvement in 1) locomotion and 2) oculomotor control is well established. Potential deficits in both, or either of these could shift the framing of this study from a social, to a motor one. The authors rightly address possible confounds related to the cerebellum's involvement in locomotion by looking at the average speed of fish during the non-stimulus period of the experiment. However, a lack of differences in speed does not necessarily mean a lack of deficit in locomotion. Analyses comparing turning behavior between control and experimental groups, such as turning frequency during the non-stimulus period, and/or turning velocity during the stimulus period (using quantile analysis) could rule out the possibility of such differences existing. Interestingly, lower average speeds in the aldoca::BoTx fish, and reelin mutants were observed by the current authors. This already serves as a potential confound for the findings on diminished social behavior in these groups of fish. It isn't inconceivable that other differences in locomotion relevant to approach and/or orienting behaviors could be found. For example, Koyama et al 2021 previously reported lower turning frequency in the cbln12::BoTx fish. If such differences exist in the granule cell inhibited fish, it could underlie the observed differences in social behavior. Hence, we think testing for such deficits is critical.

7. It would also be important to know whether cerebellar impaired fish's time spent in the ROI, and orienting behavior in the non-stimulus condition is comparable to controls. If a difference exists in the baseline condition, it could require further analyses focused on proportional changes ( eg. 1) % of time in ROI with social stimulus/ % of time in ROI without social stimulus, and 2) % of time exhibiting orienting behavior with social stimulus/ % of time exhibiting orienting behavior without social stimulus). Such differences existing at baseline could further hint at subtle locomotor related deficits.

8. Social behavior is known to be mutually dependent on the actions of individuals. Hence, we think an argument could be made for doing paired analyses of the test and stimulus fish, instead of analyzing test fish behavior alone. The aim is to see if correlations exist between test fish and stimulus fish behaviors in the present study, particularly in the cerebellar impaired fish. For example, analysis could reveal a positive correlation in percentage of time spent 1) in the ROI, 2) engaged in orienting behavior, or 3) both, between test and stimulus fish. While it would be difficult to establish a causal arrow, it could address whether a negative feedback loop exists purely based on either fish's behavior. Explicitly, if for some reason a fish exhibits repelling behavior (for example, subtle, but erratic swimming in cerebellar impaired fish, as reported in the aldoca::BoTx fish), this could cause a negative feedback loop where one fish avoids the other, eventually leading to diminished social behavior in both fishes (a case of rejecting those who reject you, and vice versa). Given the approach and orienting behavior studied here has been shown to be dependent on individual fish's engagement (Stednitz, 2018), we think this is a critical factor to address.

9. Zebrafish social behavior is influenced by size. Outside of the zebrafish/medaka experiment, the authors did not comment on whether paired fish were size matched. Further, sex is also known to influence zebrafish social behavior (including the orienting behavior studied here), but nothing was reported on sex in the current manuscript. Adding reports, or analyses ruling out the effects of size, and sex of paired fish would strengthen the claims of this study, or, at the very least, allay concerns readers may have about the influence of these factors on the social behaviors being studied- especially in fish with cerebellar deficits.

10. In various parts of the discussion, the authors seem to acknowledge that the differences in social behavior seen in cerebellar impaired fish could be driven by motor deficits. However, this interpretation is not consistent throughout the manuscript, and it vacillates between arguing for a social deficit, or a motor deficit, as the primary driver of the observed behavioral differences. The analyses suggested above may provide further evidence that motor deficits could underlie perceived social deficits in the cerebellar impaired fish. If this turns out to be true, we believe the study would still provide valuable insight into how the cerebellum indirectly influences social behaviors through impairments in motor control. Furthermore, how distinct cerebellar deficits converge on the same behavioral outcome would still be insightful. However, the authors would have to re-work the language and general theme of the manuscript to consistently frame it as observed differences in social behaviors possibly mediated through impairments in motor control.

Minor concerns:

1. To count as orienting behavior fish need to be 1) in the ROI and 2) be oriented +- 22.5 - 67.5 degrees from the direction perpendicular to the divider. A largely stationary fish could meet this orienting behavior criteria given they remain within the ROI and required angles. This, presumably, falls outside the authors' conceptual definition of orienting behavior, which suggests a more dynamic, synchronized activity, and could thus inflate orienting scores. An analysis of the fish's speed during epochs of orienting behavior could address this. While ruling out the stationary fish issue, such analysis could provide information on whether fish in the experimental group are slower/impaired in executing orienting behavior. As a minor suggestion, a plot with time on the x-axis, cumulative distance traveled on the y-axis and colored shadings representing periods of orienting, and non-orienting behavior respectively, could also prove informative to readers.

2. For clarification, outside of the zebrafish/medaka experiment, did all fish interact with another fish only once? i.e did all fish interact with the same number of fish? If not, we believe this should be reported. Some fish might have greater exposure, and hence more acclimatization to experimental conditions and/or other fish. While the latter may be less concerning for control animals (based on data in this study), it may be relevant for cerebellar impaired animals (eg. anxiety based modulation).

3. There seems to be a high level of variability in cerebellar impaired fish's behavior, with some performing well within the range of controls in some instances. Could the authors speculate on the source of this variability?

4. Was there a reason why GFP negative siblings were not used as controls in the standard/small tank experiments with cerebellar impaired animals? The current form of the experiment changes two variables, instead of one. It changes; 1) the stimulus fish (sibling vs wildtype) and 2) the tank size (which could aid if visual impairment is implicated in the behaviors). It leaves open whether cerebellar impaired fish performance would potentially improve with siblings over wildtype. This would be particularly relevant if the preference for unfamiliar fish seen the juvenile stage, reverses or abates, in adulthood.

5. If representative images of the different transgenic lines are not shown, a diagram would at least be helpful for readers to understand expression patterns.

6. Primer sequences are difficult to read in-text and could be better presented in a table.

7. In Figure 1J and 1K, there are lines connecting individual data points across different exposure times. It would be interesting to see similar lines connecting data points across no stimulus/stimulus conditions, to better visualize the change on an individual basis.

Finally, there were few minor discrepancies in the reporting of the statistics in the body of the text, versus the table:

page 21- paragraph 2, line 3- reference fig should by figure 1 H, wenot I, J

page 23 paragraph 1 line 2 (figure 2B report)- p = 0.0023 in text, but 0.0027 in the statistics table

page 23 paragraph 1 line 14 (figure 2F report)- effect size = -.56 in text but .56 in statistics table

page 24 (figure 3C report)- effect size = - 0.62 in text, but .62 in statistics table

page 25 (figure 4B report) - p = 0.34 in text, but 0.034 in table

page 25 (figure 4C report)- p = 0.0053 in text, but 0.0055 in table

page 25 (figure 4D report)- effect size = -0.34 in text, but 0.34 in table
