# Supplementary figures and images for: The Zebrafish Cerebellar Neural Circuits Are Involved in Orienting Behavior
Source: eNeuro. 2024 Oct 24;11(10):ENEURO.0141-24.2024. doi: 10.1523/ENEURO.0141-24.2024 (PMC11521796; doi:10.1523/ENEURO.0141-24.2024)

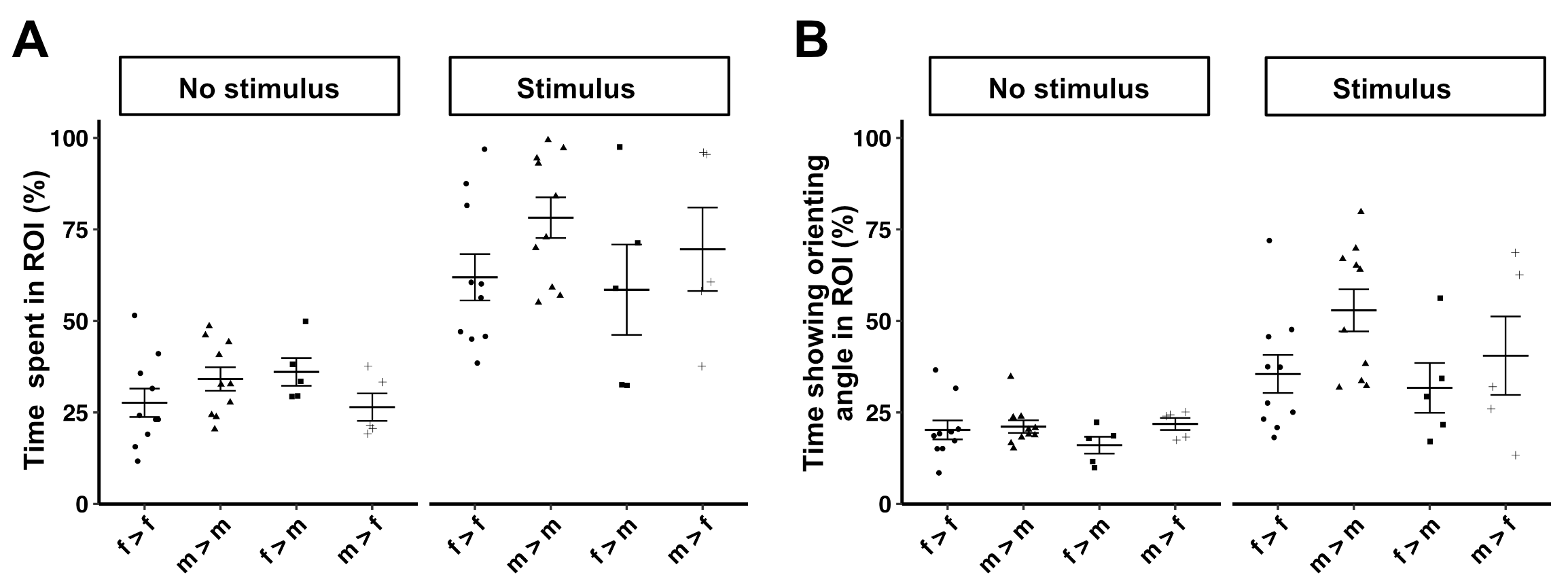

Supplement: Figure 1-1 — Sex difference on orienting behavior. Orienting behaviors were analyzed in combinations of test and stimulus fish: female and female (f > f), male and male (m > m), female and male (f > m), and male and female (m > f). Percentages of time spent in the ROI (A) and percentages of time that fish showed the orienting angles (B) were measured. The time spent in the ROI and the time that fish showed the orienting angles were not significantly different across all possible sex pairings (female and female; n = 10, male and male; n = 10, female and male; n = 5, male and female; n = 5). Download Figure 1-1, TIF file. [file eneuro-11-ENEURO.0141-24.2024-s001.tif]

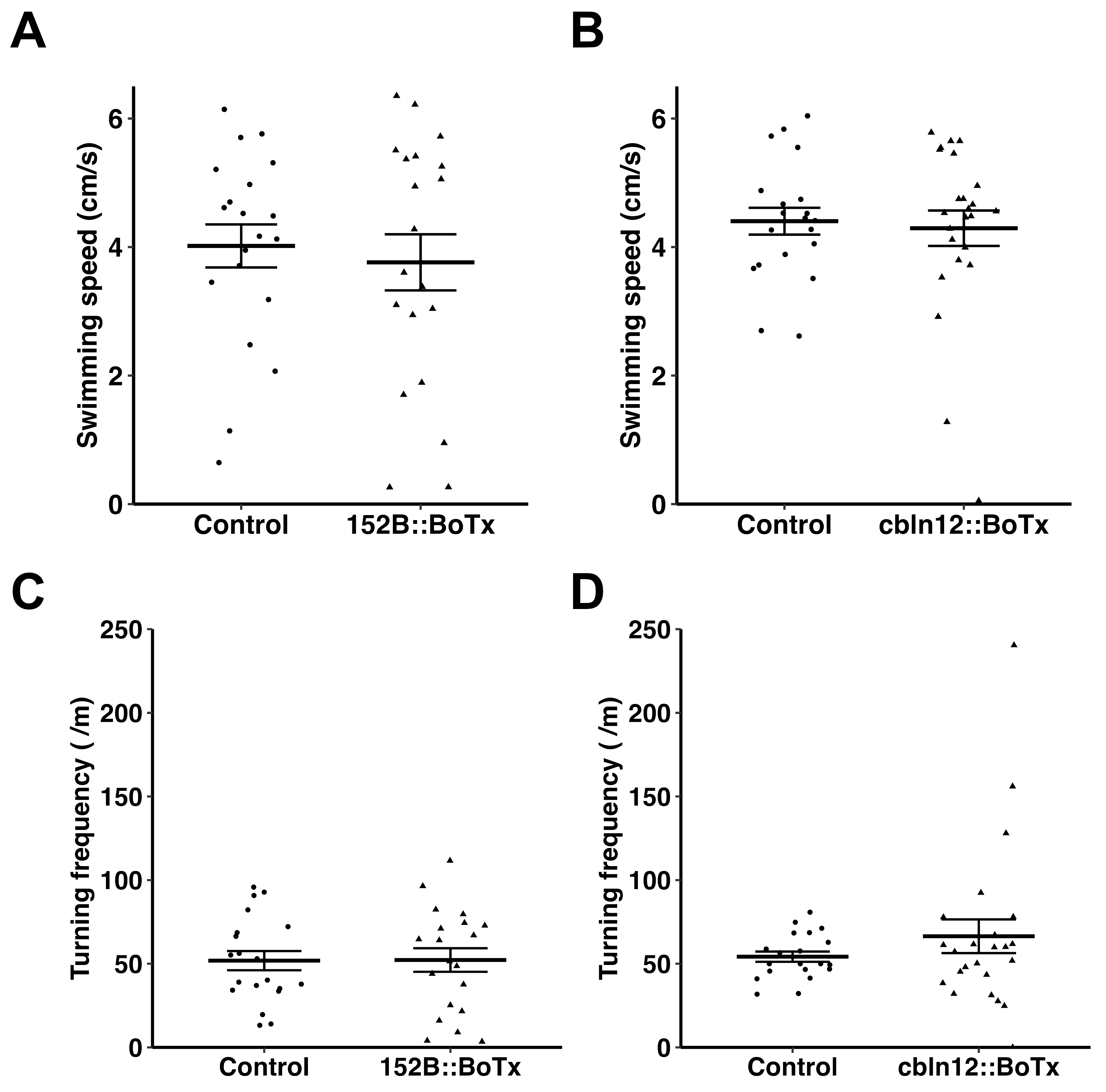

Supplement: Figure 2-1 — Swimming behavior of GC-silenced zebrafish. Swimming speed of 152B::BoTx and control (A), cbln12::BoTx and control (B). Swimming speed under no-stimulus conditions was calculated. The swimming speed was not significantly different between 152B::BoTX and control (n = 20 each), and between cbln12::BoTx and control (n = 24 and n = 20, respectively). Turning frequency of 152B::BoTx and control (C), cbln12::BoTx and control (D). Turning frequency under no-stimulus conditions was calculated. The turning frequency was not significantly different between 152B::BoTx and control (C, n = 20 each) and between cbln12::BoTx and control (D, n = 24 and n = 20, respectively). Download Figure 2-1, TIF file. [file eneuro-11-ENEURO.0141-24.2024-s002.tif]

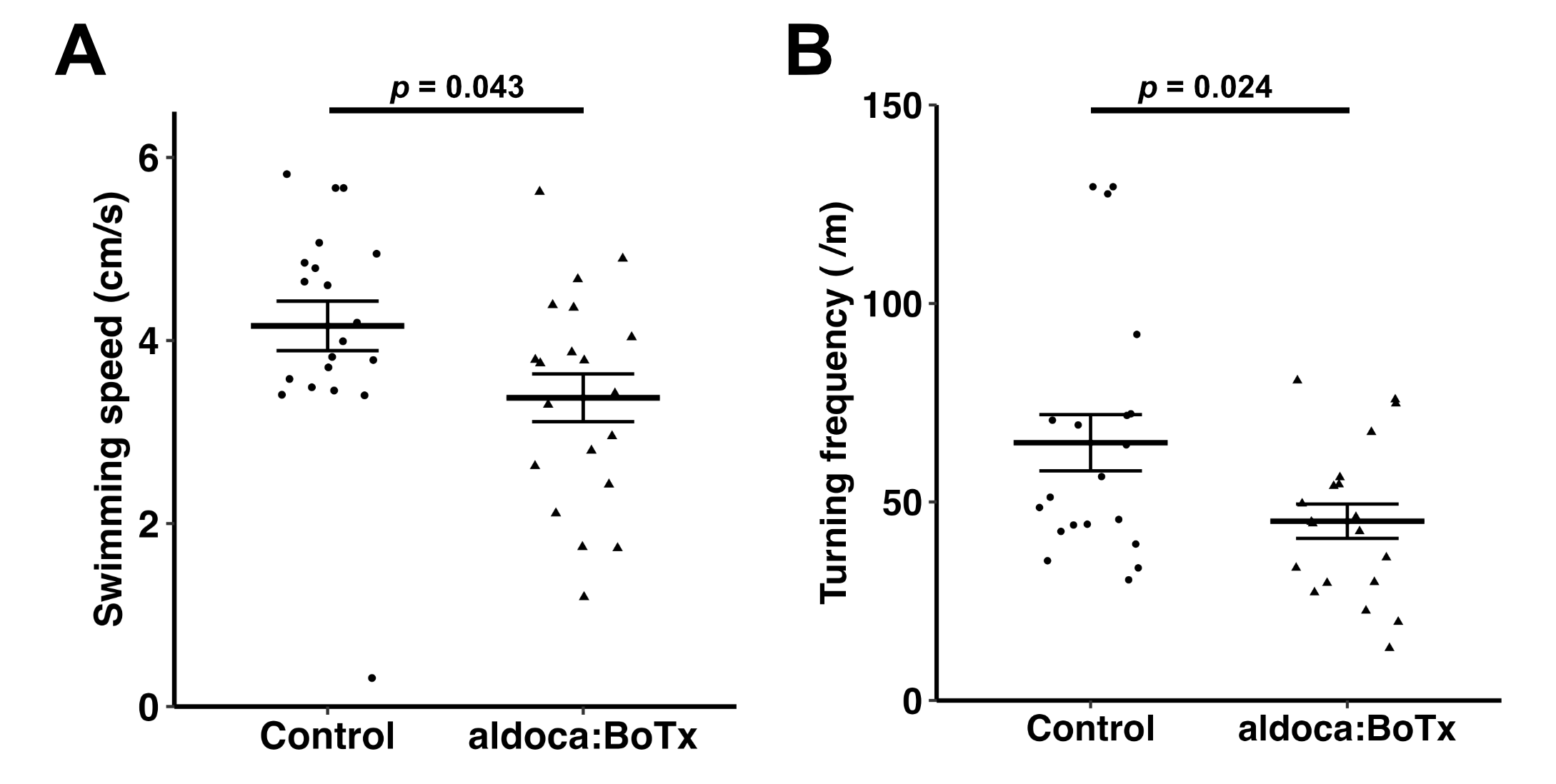

Supplement: Figure 3-1 — Swimming behavior of PC-silenced zebrafish. Swimming speed of aldoca:BoTx and control (A). Swimming speed under no-stimulus conditions was calculated. The swimming speed of aldoca:BoTx was lower than that of the control (A, n = 20 each). Turning frequency of aldoca:BoTx and control (B). Turning frequency under no-stimulus conditions was calculated. The turning frequency of aldoca:BoTx was lower than that of the control (B, n = 20 each). Download Figure 3-1, TIF file. [file eneuro-11-ENEURO.0141-24.2024-s003.tif]

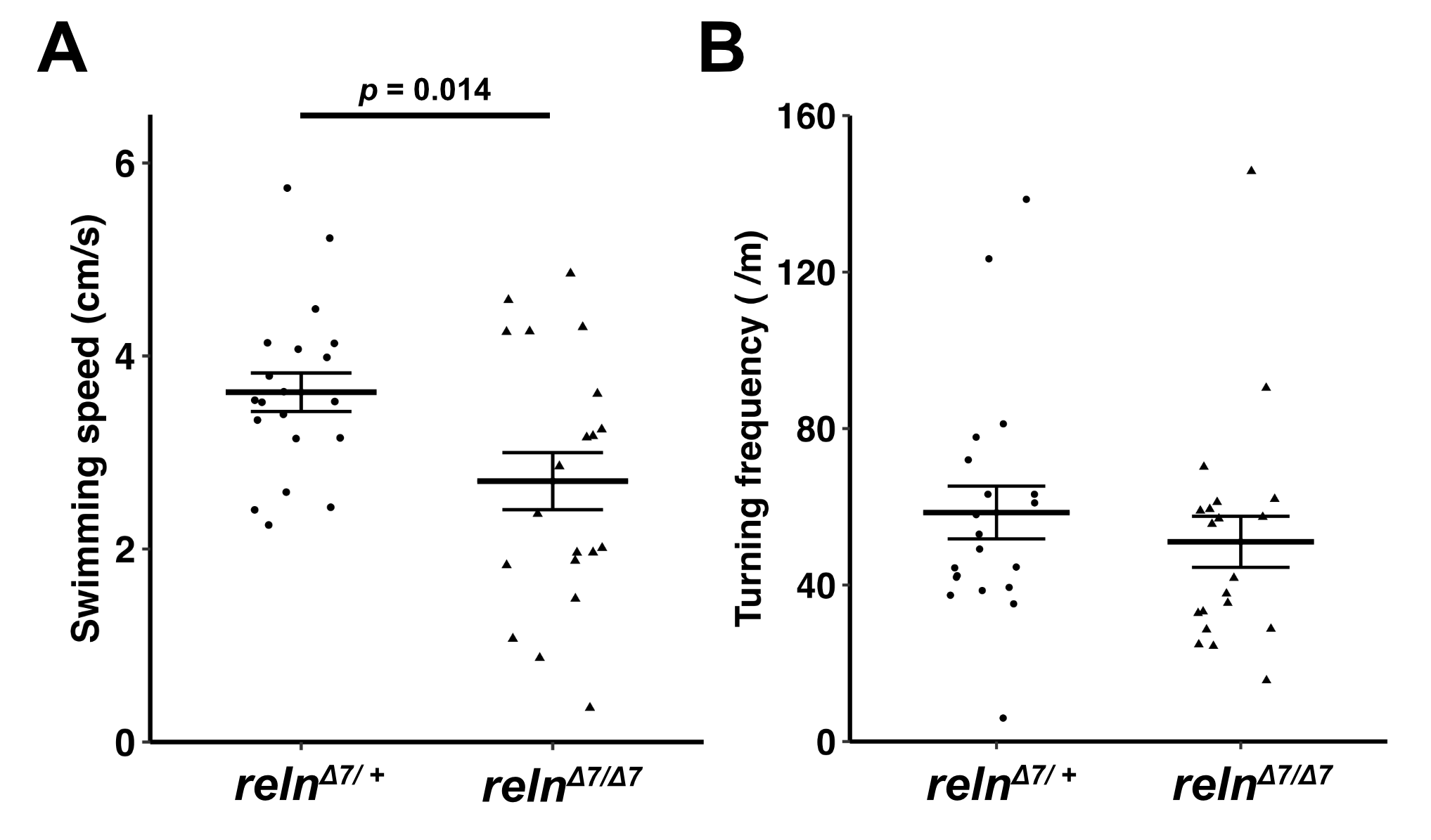

Supplement: Figure 4-1 — Swimming behavior of reln mutant zebrafish. Swimming speed of relnΔ7/Δ7 and relnΔ7/+ (A). Swimming speed under no-stimulus conditions was calculated. The swimming speed of relnΔ7/Δ7 was lower than relnΔ7/+ (A, n = 20 each). Turning frequency of relnΔ7/Δ7 and relnΔ7/+ (B). Turning frequency under no-stimulus conditions was calculated. The turning frequency was not significantly different between relnΔ7/Δ7 and relnΔ7/+(B, n = 20 each). Download Figure 4-1, TIF file. [file eneuro-11-ENEURO.0141-24.2024-s004.tif]

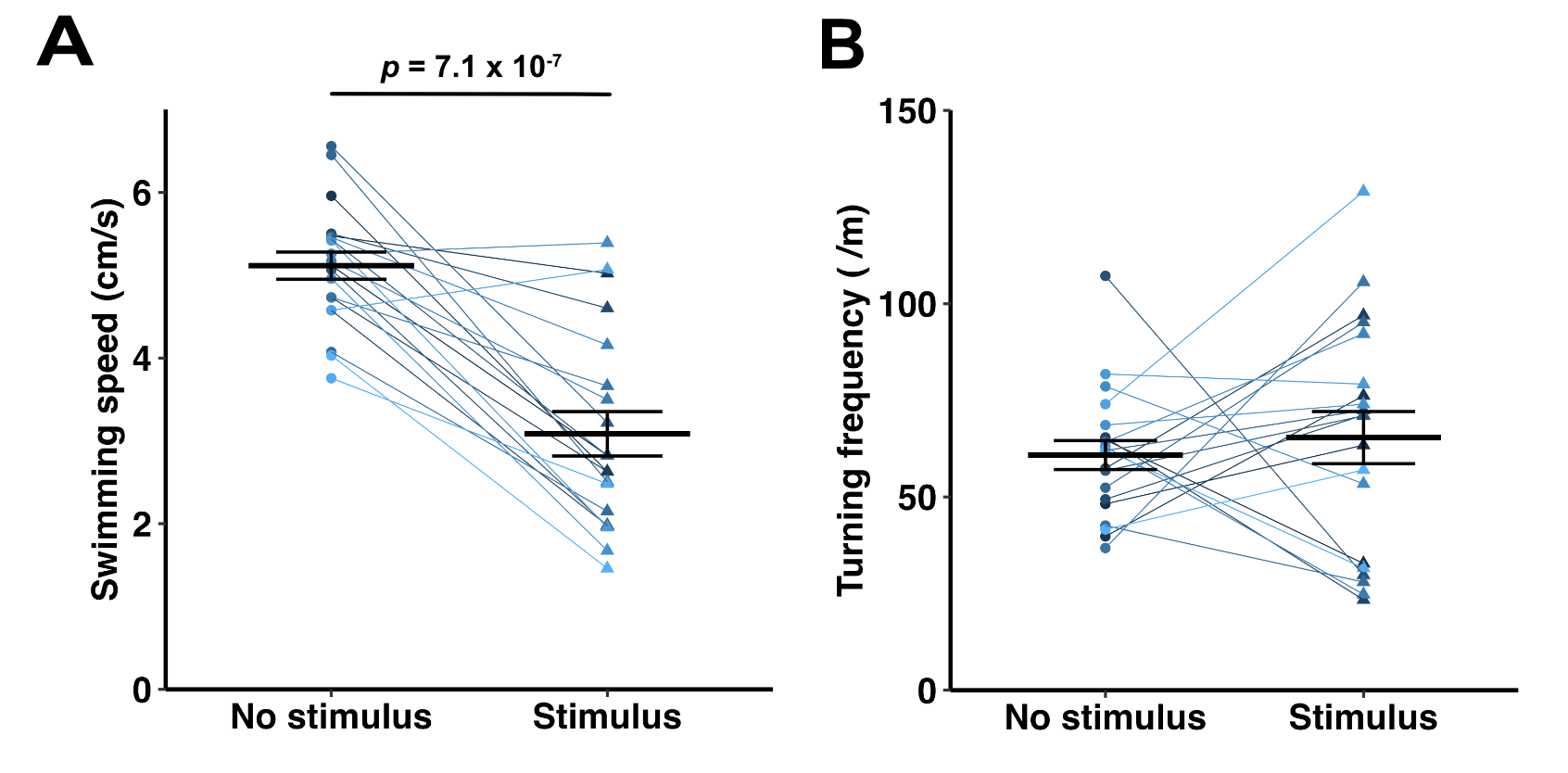

Supplement: Figure 5-1 — Swimming behavior of adult zebrafish. Swimming speed of adult zebrafish under no-stimulus or social stimulus conditions (A). Turning frequency of adult zebrafish under no-stimulus or social stimulus conditions (B). The swimming speed significantly decreased in the fish that exhibited orienting behavior under stimulus conditions, compared to no-stimulus conditions (A, n = 20). Turning frequency did not change in the fish that showed orienting behavior under stimulus conditions, compared to no-stimulus conditions (B, n = 20). Download Figure 5-1, TIF file. [file eneuro-11-ENEURO.0141-24.2024-s005.tif]
